# Photoinduced charge separation and DNA self-repair depend on sequence directionality and stacking pattern†

**DOI:** 10.1039/d3sc04971j

**Published:** 2023-12-28

**Authors:** Corinna L. Kufner, Sarah Crucilla, Dian Ding, Petr Stadlbauer, Jiří Šponer, Jack W. Szostak, Dimitar D. Sasselov, Rafał Szabla

**Affiliations:** a Department of Astronomy, Harvard-Smithsonian Center for Astrophysics 60 Garden Street Cambridge MA 02138 USA corinna.kufner@cfa.harvard.edu; b Department of Earth and Planetary Sciences, Harvard University Cambridge Massachusetts 02138 USA; c Howard Hughes Medical Institute, Department of Molecular Biology and Center for Computational and Integrative Biology, Massachusetts General Hospital Boston Massachusetts 02114 USA; d Department of Chemistry and Chemical Biology, Harvard University Cambridge Massachusetts 02138 USA; e Institute of Biophysics of the Czech Academy of Sciences Královopolská 135 61200 Brno Czech Republic; f Regional Centre of Advanced Technologies and Materials, Czech Advanced Technology and Research Institute (CATRIN), Palacky University Olomouc Slechtitelu 241/27, 783 71, Olomouc – Holice Czech Republic; g Howard Hughes Medical Institute, The University of Chicago Chicago IL 60637 USA; h Department of Chemistry, The University of Chicago Chicago Illinois 60637 USA; i Institute of Advanced Materials, Faculty of Chemistry, Wrocław University of Science and Technology Wybrzeże Wyspiańskiego 27 Wrocław 50-370 Poland rafal.szabla@pwr.edu.pl

## Abstract

Charge separation is one of the most common consequences of the absorption of UV light by DNA. Recently, it has been shown that this process can enable efficient self-repair of cyclobutane pyrimidine dimers (CPDs) in specific short DNA oligomers such as the GAT=T sequence. The mechanism was characterized as sequential electron transfer through the nucleobase stack which is controlled by the redox potentials of nucleobases and their sequence. Here, we demonstrate that the inverse sequence T=TAG promotes self-repair with higher quantum yields (0.58 ± 0.23%) than GAT=T (0.44 ± 0.18%) in a comparative study involving UV-irradiation experiments. After extended exposure to UV irradiation, a photostationary equilibrium between self-repair and damage formation is reached at 33 ± 13% for GAT=T and at 40 ± 16% for T=TAG, which corresponds to the maximum total yield of self-repair. Molecular dynamics and quantum mechanics/molecular mechanics (QM/MM) simulations allowed us to assign this disparity to better stacking overlap between the G and A bases, which lowers the energies of the key A^−^˙G^+^˙ charge transfer state in the dominant conformers of the T=TAG tetramer. These conformational differences also hinder alternative photorelaxation pathways of the T=TAG tetranucleotide, which otherwise compete with the sequential electron transfer mechanism responsible for CPD self-repair. Overall, we demonstrate that photoinduced electron transfer is strongly dependent on conformation and the availability of alternative photodeactivation mechanisms. This knowledge can be used in the identification and prediction of canonical and modified DNA sequences exhibiting efficient electron transfer. It also further contributes to our understanding of DNA self-repair and its potential role in the photochemical selection of the most photostable sequences on the early Earth.

## Abstract 2

The exposure of DNA to UV light may result in electron transfer between the bases. It can entail self-repair of common DNA lesions and the efficiency of these two processes is controlled by stacking overlap of the nucleobases.

## Graphical abstract

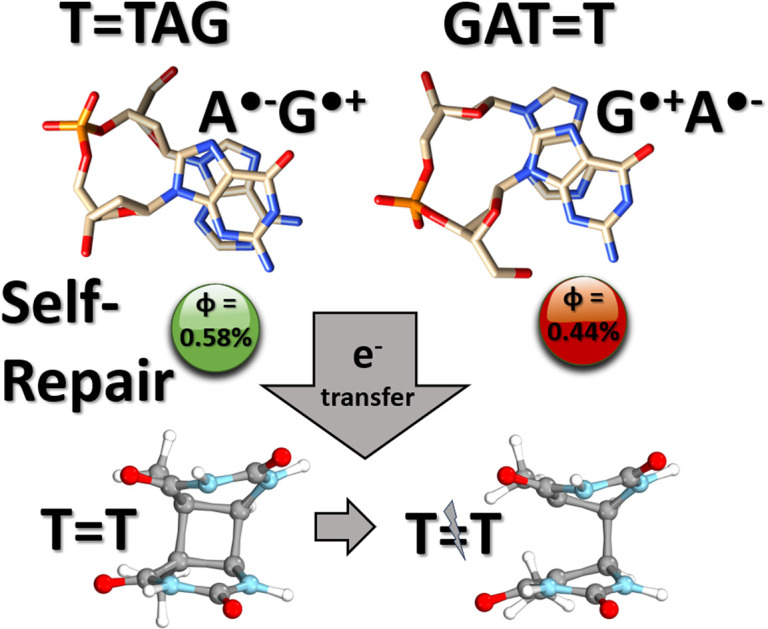

## Introduction

Photoinduced charge separation is one of the main phenomena occurring in nucleic acids during the exposure to ultraviolet (UV) light.^1^ Despite its far reaching implications for biochemistry, biology and even materials sciences,^2–5^ the key experimental proof of photoinduced charge separation in native unmodified DNA was delivered less than 20 years ago. This experiment involving transient absorption spectroscopy demonstrated that UV excitation of polydeoxyadenosine could populate long-lived electronic states, which were assigned to charge transfer (CT) between the neighbouring stacked adenine nucleobases.^6^ While numerous examples of CT states have been corroborated in different modified and native forms of DNA since then,^1,7–14^ the key factors controlling the efficiency of this process still remain obscure. Consequently, prediction of DNA sequences capable of performing efficient UV-induced charge transfer is still a challenge.

Among different processes that can be triggered by charge separation in DNA, self-repair of cyclobutane pyrimidine dimers (CPDs) attracted substantial attention, recently.^15,16^ CPDs are the most frequently formed photolesions during the exposure of DNA to ultraviolet light and their most characteristic structural element is the cyclobutane ring formed between two adjacent pyrimidine bases.^17–21^ Formation of this cyclobutane ring affects the structure of the sugar–phosphate backbone and precludes biochemical activity such as DNA replication and transcription.^21,22^ In biology, CPD repairing enzymes, such as photolyases, repair the lesions through the injection of an electron from the flavin adenine co-factor, after the absorption of visible light.^23–27^ Similarly, specific DNA sequences or alternative nucleobases were shown to trigger nonenzymatic DNA self-repair *via* photoinduced electron transfer.^16,28–30^ The most prominent examples of DNA self-repair were demonstrated for the damaged GAT=T sequence (“=” representing the CPD) and for 2,6-diaminopurine (D) and 8-oxoguanine (O) nucleobases located in the vicinity of CPDs.^31,32^ In particular, the GAT=T sequence was described to undergo sequential electron transfer from guanine upon its photoexcitation.^33–35^ In other words, the yields of nonenzymatic DNA self-repair are a manifestation of how efficiently photoinduced charge separation can occur in specific DNA sequences and whether lifetimes of the CT states are sufficiently long to invoke a photochemical reaction.

It is worth emphasizing that highly efficient self-repair of CPDs greatly improves the photostability of specific sequences and was also suggested as a possible selection factor for primordial RNA and DNA oligomers from the rich pool of random sequences.^1,15,36,37^ More importantly, UV light has been proposed as one of the key sources of energy for selective prebiotic syntheses of nucleotides.^38–46^ This resulted in the consideration of the above mentioned D and O nucleobases as potential components of first informational polymers owing to their improved electron-donating and CPD-repairing properties when compared to canonical nucleobases.^31,32,47^ In particular, DNA trinucleotides containing a D nucleobase and a T=T dimer were shown to repair the CPD with yields reaching up to 92% when irradiated at 280 nm and, thus, D could protect DNA oligomers from photodamage under prebiotic conditions.^31^

As shown by Bucher *et al.*^1^ the direction of charge transfer in stacked DNA is controlled by the oxidation potentials of the nucleobases. However, the efficiency with which excited CT states are populated seems to depend on much more subtle aspects related to the local environment of the sequence of stacked bases.^2^ This could be either governed by the spatial overlap of the neighbouring stacked bases as well as by the lifetimes of the different electronic states involved in photoinduced charge separation.^31,48^ More specifically, as suggested for DT=T and T=TD trimers, a possible factor which could enable funneling the excited-state population to a CT state is the inacessibility of efficient direct photorelaxation channels of the locally excited (LE) state on D *via* an S_1_/S_0_ conical intersection.^31^ Here, we present a surprising example of the damaged T=TAG oligomer (5′-end denoted first), which exhibits self-repair quantum yields which are higher by ∼30% than in the case of the equivalent GAT=T oligomer with opposite sequence direction when exposed to UV irradiation at 285 nm. Since this difference cannot be explained by the ordering and oxidation potentials of nucleobases, we provide a mechanistic rationale for this process based on molecular dynamics simulations, quantum mechanics/molecular mechanics (QM/MM) calculations and explorations of excited-state potential energy surfaces using the algebraic diagrammatic construction to the second order method [ADC(2)].^49,50^ We show that in contrast to GAT=T, direct photorelaxation of the LE and CT intermediate states of the photoexcited T=TAG tetramer is substantially hindered, which enhances sequential electron transfer between stacked bases and enables efficient and selective CPD self-repair.

## Results and discussion

### Experimental observation of higher self-repair quantum yields in T=TAG when compared to GAT=T

**Fig. 1:**
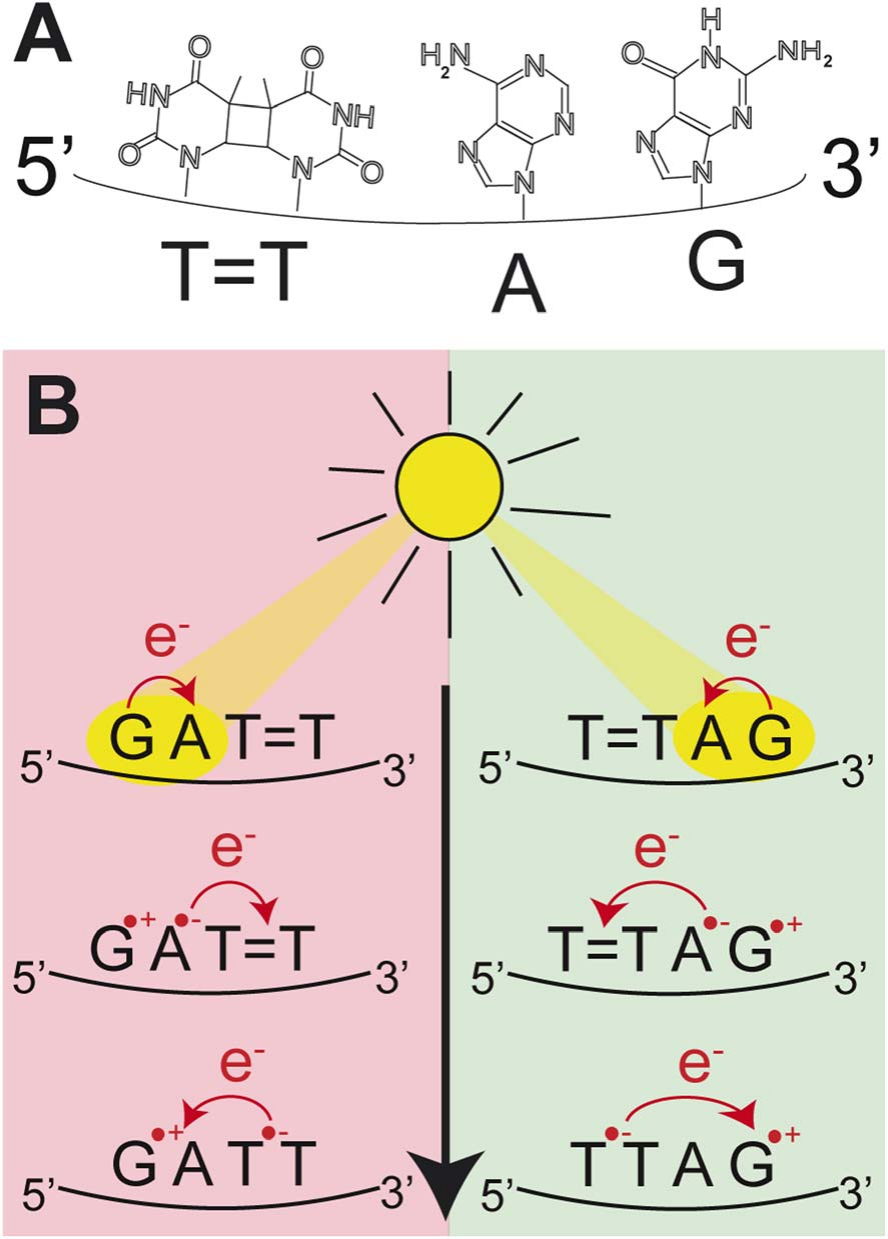
(A) Molecular structures of the nucleobases in the DNA sequence T=TAG. (B) Schematic representation of the CPD self-repair mechanism promoted by photoinduced sequential electron transfer in the DNA sequences, GAT=T and T=TAG.

**Fig. 2:**
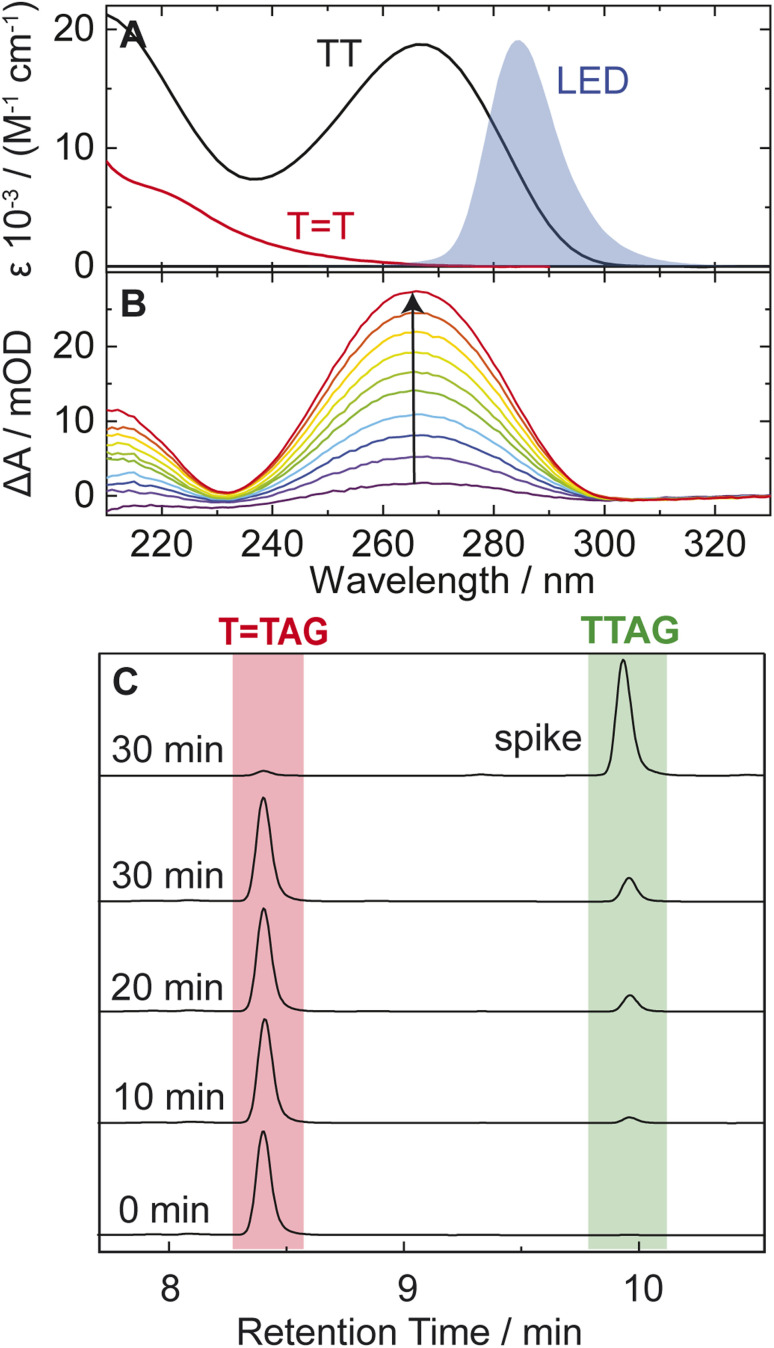
(A) Molar decadic absorption coefficients of the T=T CPD lesion (red) and the undamaged TT dinucleotide (black). The emission spectrum of the LED centered around 285 nm, which was used for irradiation, is shown as blue shade for comparison. (B) UV absorption difference spectra of a 30 μM solution of the sequence T=TAG after increasing times (1–10 min) of exposure to 285 nm irradiation with an average power of 0.36 mW. The recovery of the 266 nm absorption (arrow) is indicative of the self-repair to TTAG. (C) Analytical HPLC analysis of the sequence T=TAG (8.4 min) after different irradiation times. Upon irradiation a recovery of the undamaged sequence TTAG (9.9 min) can be observed. The chromatogram on top is spiked with undamaged TTAG for reference.

In our previous works, we demonstrated the self-repair of the short single-stranded DNA sequence, GAT=T, *via* photoexcitation at 290 nm.^33^ After absorption of a UV photon, the GAT=T molecule sequentially populates minima on the potential energy surface of the lowest excited electronic state (S_1_ minima), which can be associated with the G^+^˙A^−^˙T=T and G^+^˙AT=T^−^˙ charge transfer states (Fig. 1 left).^14,35^ These states can be accessed right after the photoexcitation of the G base, which is responsible for the majority of the absorption of the oligomer at wavelengths longer than 280 nm.^14,30^ Here, we performed comparative UV irradiation experiments of the DNA oligonucleotides T=TAG and GAT=T (Fig. 1). Exposure of the sequences to long-wavelength irradiation from an LED, centered around 285 nm (average power at the sample position 0.36 mW), allowed us to excite the Guanine (predominantly) and Adenine (partially) bases and minimized photoreversal from direct absorption of the T=T dimer (Fig. 2A and S7†).^51,52^ As presented in Fig. 2B, continuous UV-irradiation of an aqueous T=TAG solution (pH 6.9, ∼30 μM) in our system lead to an absorbance increase of several 10 mOD at 266 nm within 10 minutes. The irradiation induced difference spectra clearly correspond to typical absorption pattern exhibited by thymine bases in DNA, which is strongly indicative of the self-repair process (Fig. 2A). Complementary HPLC analysis corroborated this finding (Fig. 2C). The chromatogram of the damaged sequence T=TAG as starting material (bottom) shows a peak at 8.4 min. Upon exposure to 285 nm light, the undamaged sequence TTAG gradually recovers, visible as an increase in absorption at 9.9 min. After 30 min of irradiation, the irradiated sample was spiked with the undamaged sequence TTAG for reference (top chromatogram). The emergence of one single peak at 9.9 min confirms the self-repair to TTAG. The corresponding chromatograms for the sequence GAT=T are shown in Fig. S1 in the ESI.†

**Fig. 3:**
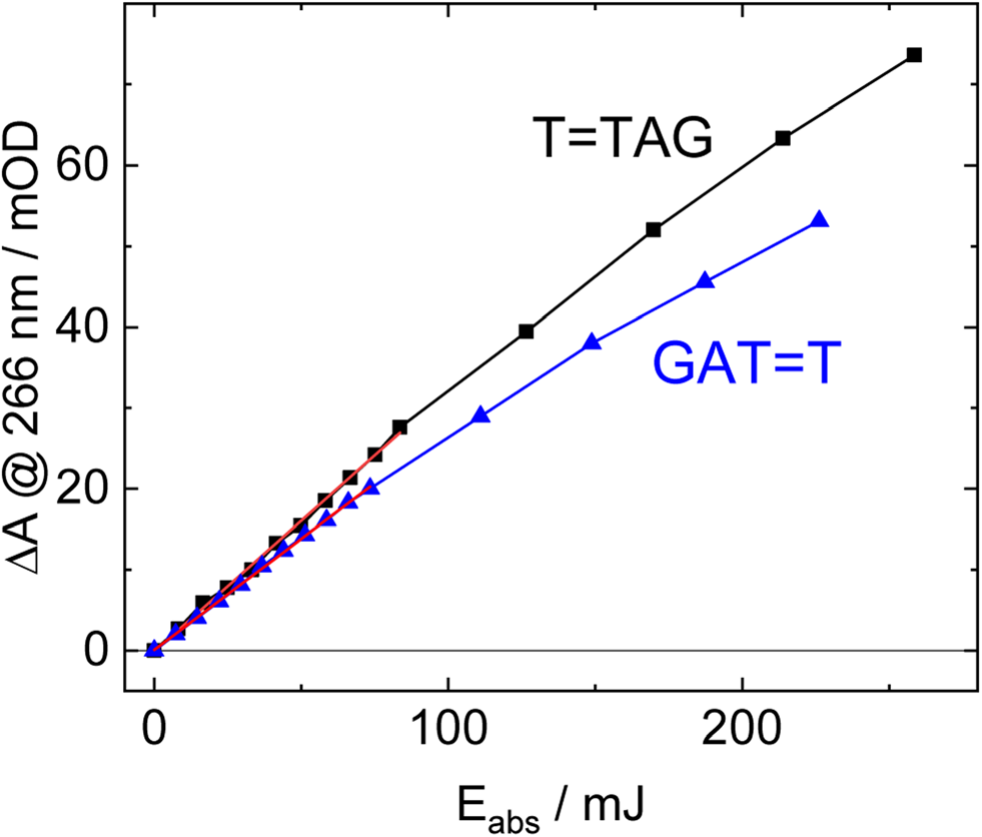
Absorbance difference spectra of T=TAG (black) and GAT=T (blue) at 266 nm as a function of absorbed light dose at 285 nm. The data at low doses can be fitted with a linear trend line (red). The slope of the trend line is linearly proportional to the quantum yield of the self-repair.

**Fig. 4:**
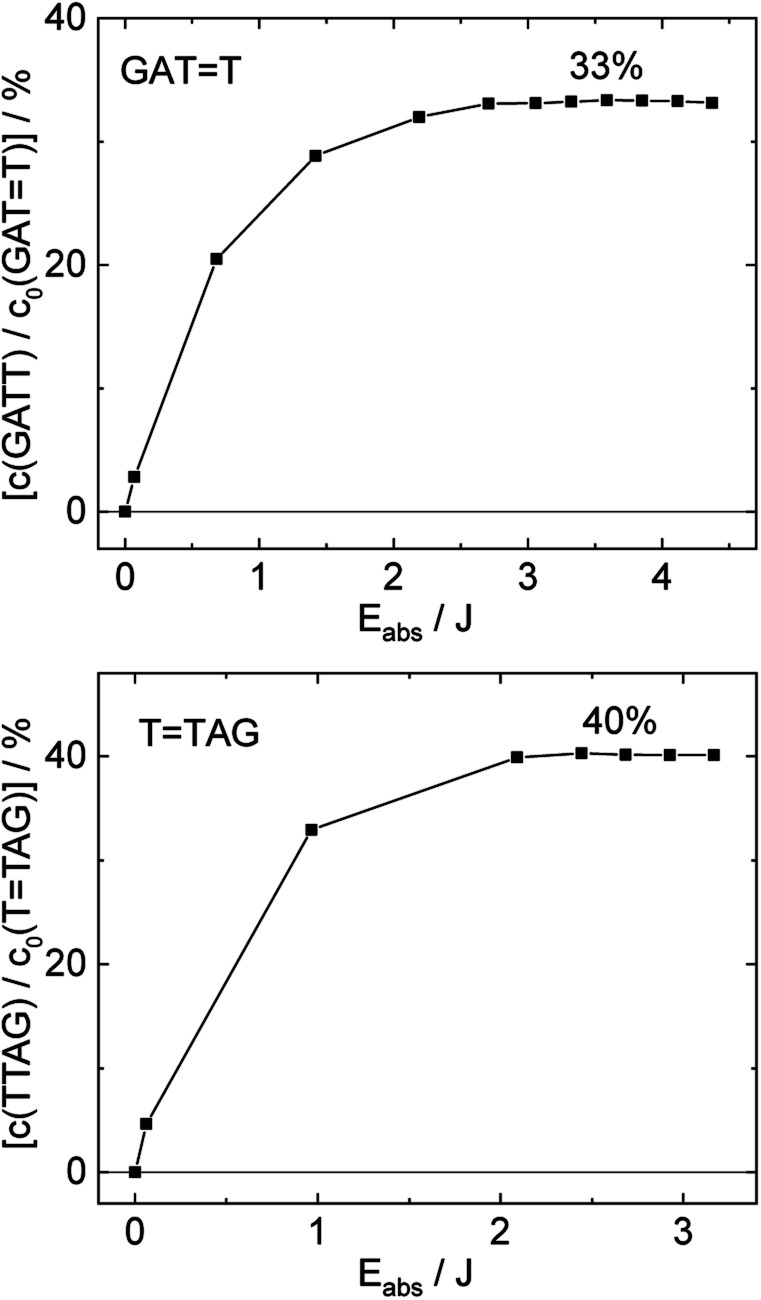
Concentration of the undamaged sequence GATT (top) and TTAG (bottom) divided by the initial concentration of the damaged sequence GAT=T (top) and T=TAG (bottom) as starting material as a function of absorbed photon dose at 285 nm. In case of the GAT=T sequence a photostationary equilibrium between repair and damage formation is reached after absorption of 3.5 J at a level of ∼33%. The sequence T=TAG reaches the equilibrium earlier after absorption of 2.5 J at a level ∼40%. The absorption coefficients at 266 nm were taken from Pan *et al.*^30^

The increase in absorbance at 266 nm due to the recovery of undamaged TTAG from T=TAG (black arrow in Fig. 1B) can be plotted as a function of the photon dose absorbed by the molecules (Fig. 3). At low irradiation doses, the increase in absorbance at 266 nm is linear. The data in this range can be fitted with a linear trendline (red). The slope of the initial absorbance increase is linearly proportional to the quantum yield of the CPD self-repair (see ESI† for details). The quantum yield of CPD self-repair was found to be 0.44 ± 0.18% for GAT=T (blue) and 0.58 ± 0.23% for T=TAG (black), respectively. At higher absorbed doses, the slope of both plots decreases. This is largely the result of approaching the photostationary state of equilibrium, but can be also attributed to the formation of secondary products, which lower the yield of the net reaction. After absorption of high irradiation doses, a photostationary equilibrium between net damage formation and self-repair can be reached (Fig. 4). In case of the sequence GAT=T, the equilibrium is reached after the absorption of 3.5 J at a level of 33 ± 13% self-repair. This result is higher than the previously reported 25% at 290 nm irradiation as well as the corresponding quantum yields.^33,53^ The differences can be attributed to the 15 nm broad LED light source, in comparison to the previously used narrowband (3 nm broad) 290 nm excitation, as self-repair quantum yields and direct photoreversal may be higher at lower wavelengths. In case of T=TAG, the equilibrium is reached only after absorption of 2.5 J at a higher level of 40 ± 16%. The error bars were estimated to 40% of the provided values according to the previous work by some of us were an analogous experimental setup was used.^54^ These results indicate that the ratio of the self-repair *vs.* the net rate of CPD formation is higher in case of T=TAG when compared to GAT=T. The photostationary equilibrium indicates the maximum total yield of the repair in UV-rich environments, under persistent exposure to irradiation.

### Structural differences between the T=TAG and GAT=T tetramers

**Fig. 5:**
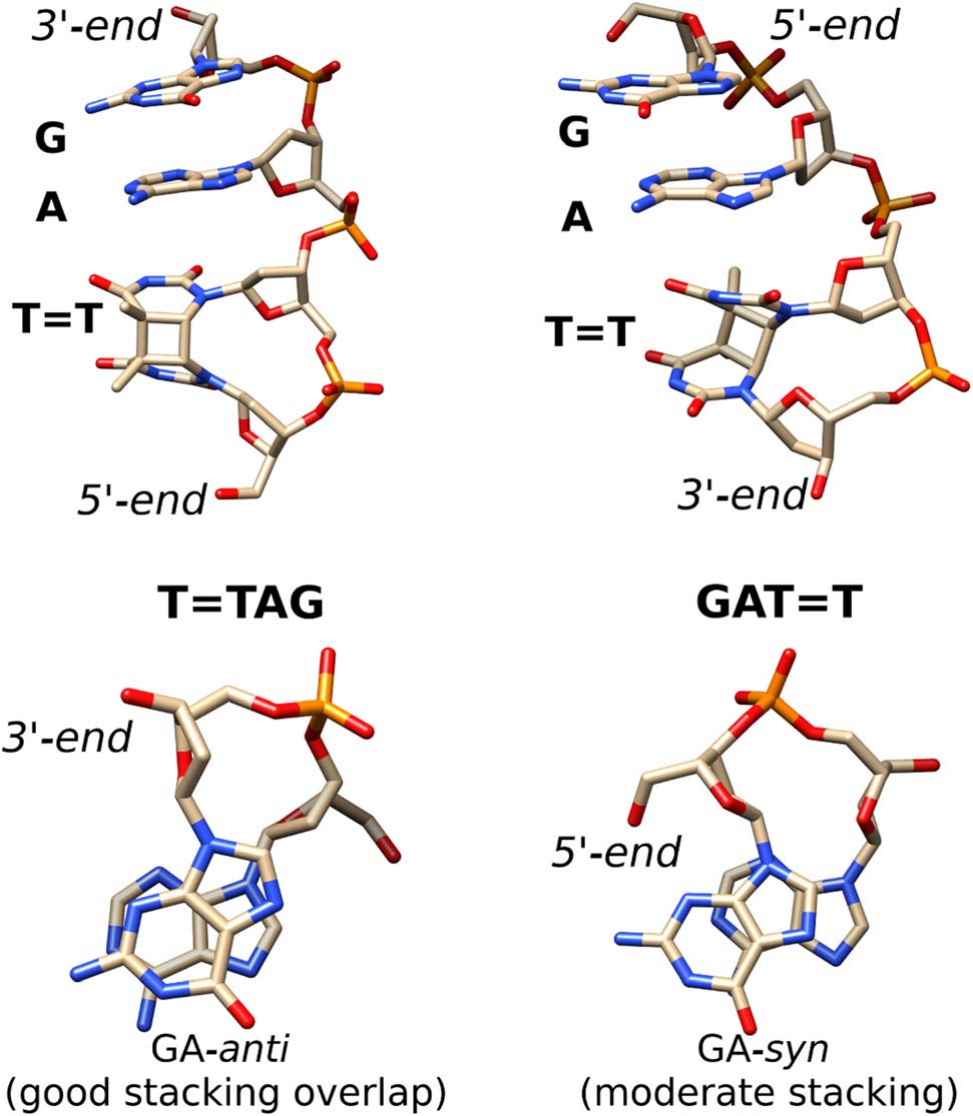
Averaged structures of the major stacked conformers obtained from the classical MD simulations of T=TAG (left) and GAT=T (right)^35^ tetranucleotides. The stacking of the G and A bases is presented at the bottom.

We further performed classical molecular dynamics and quantum chemical simulations, to provide mechanistic rationale explaining this substantial difference in self-repairing activity between the T=TAG and GAT=T tetramers. Since we previously performed such calculations for the GAT=T tetranucleotide, here, we applied an analogous computational protocol to the T=TAG tetramer.^35^ The overall lengths of the trajectories of our force-field based molecular dynamics simulations for T=TAG amounted to 10 μs per tetranucleotide. We selected two of the most representative stacked conformers, which have the highest contribution to the overall conformational space of T=TAG (see the ESI† for more details). The AG-*anti* conformer of T=TAG discussed in the main article is shown in Fig. 5. We used their averaged MD structures for subsequent QM/MM simulations in order to first optimize the ground-state geometry of the tetramer using density functional theory with dispersion correction (PBEh-3c functional) and next calculate their photophysical and photochemical properties.

Notably, as shown in Fig. 5, the highest populated stacked conformers of T=TAG and GAT=T differ by the relative spatial orientation of the nucleobases and the stacking patterns. The positioning of the G and A bases at the 3′-end of the T=TAG tetramer results in the two nucleobases predominantly populating the *anti* orientations with respect to the sugar scaffold. This entails a very good overlap of the six-membered counterparts of the purine rings and partial overlap of the five-membered subunits for most of the populated conformers. While the T=TAG conformer presented in Fig. 5 was populated in 9% of the simulation time, we also observed this AG-*anti* arrangement for conformers with partially unstacked or overhanging T=T dimer. Overall, the AG-*anti* arrangement was present in >50% of sampled conformations of T=TAG (see Fig. S4 in the ESI† to this article). In contrast, the G base of the GAT=T tetramer prefers to form a hydrogen bond between its N3 atom and the free 5′-OH group of the sugar, which is accompanied by the *syn* orientation of the nucleobase with respect to the sugar ring.^35^ As observed previously, the neighbouring adenine of GAT=T also prefers the *syn* orientation to maintain better stacking and the resulting GA-*syn* stacked conformer was determined to be dominant (populated in over >30% of conformations).^35^ It is worth noting though that the G and A bases of this conformer are somewhat displaced with respect to one another and less favorably stacked, having only the five- and six-membered counterparts of G and A bases stacked. As previously indicated for long-range electron transport in DNA, the degree of stacking and conformational arrangement could strongly affect the rate and yield of CT along the stack.^2^ Therefore, these structural differences are the first indication that disparities between the photochemistry of the two tetranucleotides should be expected.

### Photophysical properties of T=TAG

According to the vertical excitation energies computed for the T=TAG tetramer (see the ADC(2)/MM results Table 1), the charge transfer states can be found in the higher energy range of the UV absorption spectrum, whereas the lower energy range of the spectrum is dominated by local ππ* excitations of the purine bases and nπ* excitations of the T bases constituting the T=T dimer. The lowest-energy CT state in the Frank–Condon region involves an electron transferred from G to A and can be identified as the S_6_ state with the 
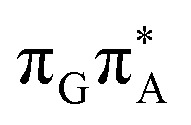
 molecular orbital character. The analogous 
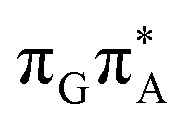
 CT state of the GA-*syn* stacked conformer of GAT=T has the vertical excitation energy higher by 0.2 eV and was identified as the S_10_ state. This demonstrates that the stacking overlap between the G and A bases can strongly affect the ordering of the electronic states and the energies of CT excitations.

**Table 1:** Vertical excitations energies (in eV) of the T=TAG tetranucleotides obtained at the ADC(2)/TZVP level of theory, assuming the ground-state minimum-energy geometry optimized with the PBEh-3c method. The results for the GAT=T tetranucleotide were taken from ref. 33 and obtained with the same approach

State/transition		*E*_exc_ [eV]	*f*_OSC_	*λ* [nm]
**AG-*anti* conformer of T=TAG**				
S_1_(LE)	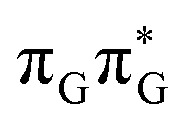	4.95	0.097	250.7
S_2_(LE)	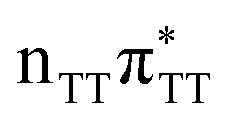	5.07	1.04 × 10^−3^	244.5
S_3_(LE)	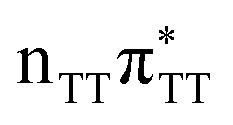	5.12	1.65 × 10^−3^	242.0
S_4_(LE)	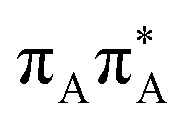	5.15	0.070	240.7
S_5_(LE)	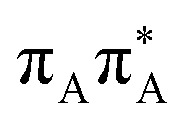	5.28	8.06 × 10^−3^	234.7
S_6_(CT)	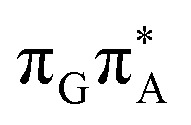	5.40	0.388	229.4

**GA-*syn* conformer of GAT=T**				
S_4_(LE)	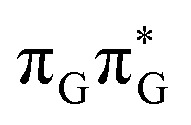	5.00	0.056	248.0
S_7_(CT)	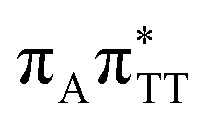	5.32	4.25 × 10^−4^	233.1
S_10_(CT)	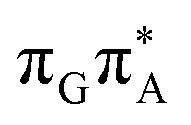	5.60	0.015	221.4

We previously identified another CT excitation in the Franck–Condon region of the GAT=T tetramer, namely the 
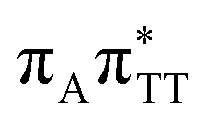
 CT state associated with an electron transferred between the A base and the T=T dimer. In fact, this excitation is the lowest energy CT state found for the ground-state geometry of the GA-*anti* conformer of GAT=T, and was identified as the S_10_ state with the excitation energy of 5.81 eV. In the case of the GA-*syn* conformer of the GAT=T tetranucleotide, the 
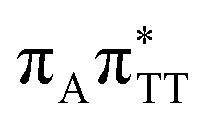
 CT state was found to be the S_7_ state with the vertical excitation energy of 5.32 eV. Nevertheless, we did not find this electronic state among the ten lowest vertical excitations of the T=TAG tetranucleotide. Furthermore, low self-repair yields of canonical trinucleotides containing CPDs was ascribed to the limited accessibility of the 
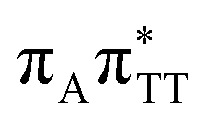
 CT state.^30,31^ Therefore, as proposed for the GAT=T tetranucleotide, we postulate that the initial photoinduced charge separation event in T=TAG is initiated with the population of the 
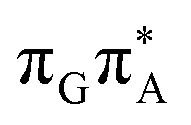
 state outside of the Franck–Condon region. The details of this mechanism are discussed in the following section.

### Sequential electron transfer in the T=TAG tetranucleotide

**Fig. 6:**
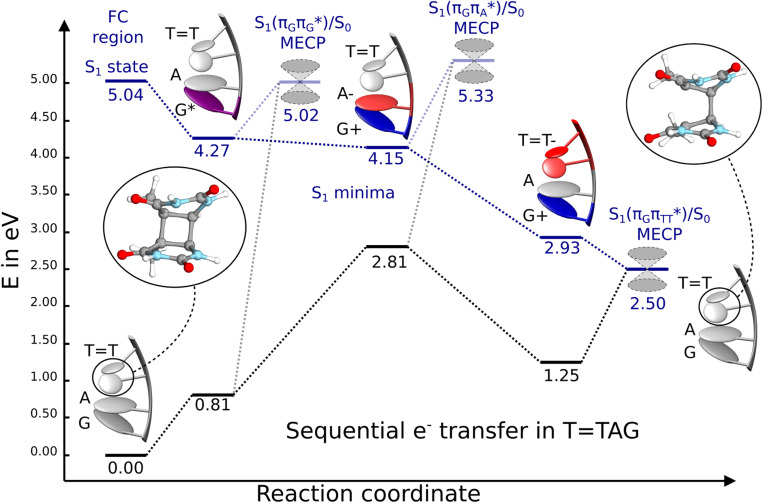
Energy diagram showing the sequential electron transfer mechanism initiated with the photoexcitation of the guanine base of the T=TAG tetranucleotide to the S_1_(
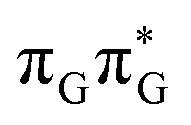
) state in the Franck–Condon (FC) region (left-hand side of the diagram). The leftmost structure corresponds to the ground-state geometry of the AG-*anti* conformer of the T=TAG tetramer. The three middle energy levels are associated with the key S_1_ minima. The partly transparent pathways leading to S_1_/S_0_ MECPs demonstrate the possible competing direct photorelaxation mechanism from the G* and A^−^˙G^+^˙ intermediate states. The rightmost structure is associated with the key S_1_/S_0_ MECP responsible for C5–C5 bond breaking. The energies were obtained with the QM_bases_/MM setup at the ADC(2)/def2-SVP level of theory (see the Computational Methods section in the ESI† for more details).

According to the calculations of vertical excitation energies, irradiation of the T=TAG tetranucleotide at 285 nm primarily results in the population of the S_1_(
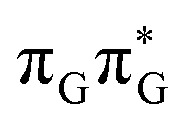
) state of the G base. We argue that subsequent electron transfer events will occur on the hypersurface of the lowest excited singlet state involving changes of molecular orbital character until the key S_1_/S_0_ state crossing is reached. Such a mechanism leading to partial splitting of the cyclobutane ring is presented in Fig. 6 and involves three intermediate S_1_ minima. Therefore, the presented self-repair mechanism of T=TAG is analogous to the sequential electron transfer (SET) process described for the GAT=T tetranucleotide.^35^ However, we identified the key differences in the SET mechanism for the GAT=T and T=TAG tetranucleotides which could further explain the higher self-repair yields found for T=TAG.

**Fig. 7:**
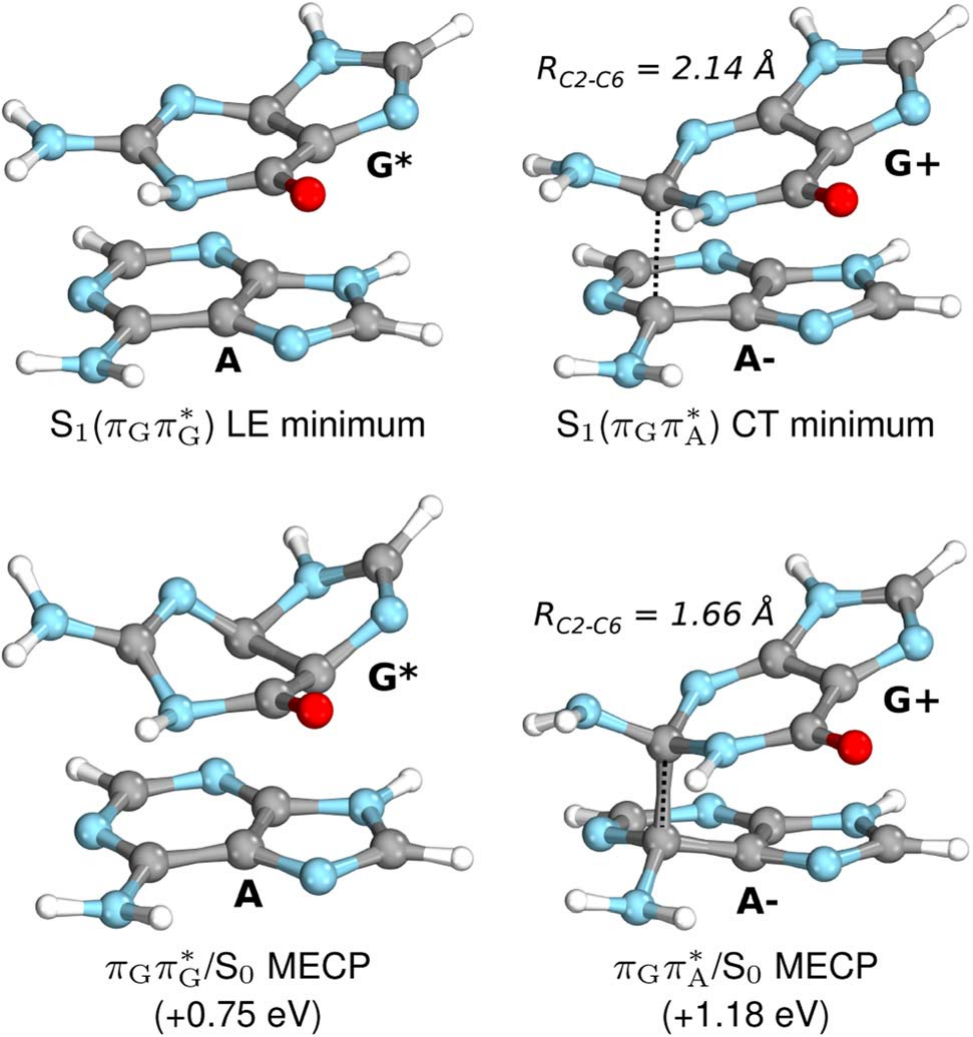
Geometries of the G and A bases located for the intermediate excited-state (S_1_) G* and A^−^˙G^+^˙ minima (top) and the geometries of the corresponding S_1_/S_0_ MECPs. The presented geometries and energies were obtained with the QM_bases_/MM setup at the ADC(2)/def2-SVP level of theory (see the Computational Methods section in the ESI† for more details).

Initial vibrational relaxation of the 
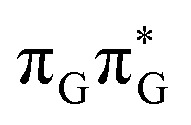
 state results in modest puckering of the aromatic ring of guanine and reaching the vicinity of the first S_1_ minimum that participates in the SET mechanism. This minimum is denoted as G* in Fig. 6 and lies 4.27 eV above the ground-state structure of the AG-*anti* conformer of T=TAG. Similarly as in the case of GAT=T, ring puckering is most pronounced at the C4 and C5 atoms and greater out of plane distortion of the C4 and N3 atoms and rotation about the C4=N3 bond leads to the S_1_(
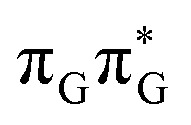
)/S_0_ minimum-energy crossing point (MECP) lying ∼0.75 eV above the G* S_1_ minimum (see Fig. 6 and 7). Considerably sloped topography of this state crossing and the associated energy barrier hinder the direct photorelaxation of the G base. We anticipate that this will increase the importance of the competitive forward electron transfer process from the G base to the neighbouring A base. It is worth noting that the analogous S_1_(
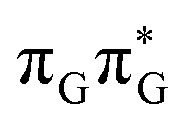
)/S_0_ MECP of the of GAT=T tetranucleotide was reported to lie only 0.15 eV and 0.50 eV above the S_1_(G*) minima for the GA-*syn* and GA-*anti* conformers, respectively. This indicates that direct photorelaxation of the G base should be more efficient in the GAT=T tetranucleotide, whereas UV-excited T=TAG tetramer should more easily undergo charge separation.

Subsequent excited-state electron transfer from the G base may allow to reach the vicinity of the A^−^˙G^+^˙ S_1_ minimum. This entails structural changes in the ring-puckering pattern of the G base, with most pronounced pyramidalization of the C2 atom and additional pyramidalization of the C6 atom of A. Consequently, the two pyramidalized C atoms create the main contact between these purine bases in the A^−^˙G^+^˙ CT minimum. This S_1_ minimum lies merely 0.12 eV below the G* minimum of T=TAG AG-*anti* conformer, which implies a weaker driving force for e^−^ transfer than in the case of the GAT=T tetramer (Δ*E* = −0.9 eV for the GA-*anti* conformer). While driving force is an important component of the electron rate within the Marcus model, the efficiency of photoinduced CT between stacked nucleobases is also dependent on the excited-state lifetime of the donor state. Given that, the direct photorelaxation of the G base is hindered in major conformers of T=TAG, we expect the G to A electron transfer process to be an important contributor to the photochemistry of this tetranucleotide.

Similarly as in the case of the 
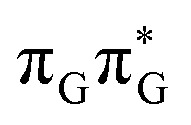
 state, the direct photorelaxation from the A^−^˙G^+^˙ minimum is hindered owing to very high energy of the S_1_(
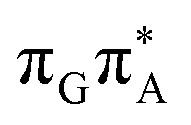
)/S_0_ state crossing, which lies 1.18 eV above the corresponding S_1_ minimum. The energy of this state crossing is even higher than the vertical excitation energy of lowest optically bright state of the G base. This state crossing involves formation of a (transient) covalent bond between the C2 atom of G and C6 atom of A. Consequently, the second electron transfer event from the radical anion of the A base to the T=T dimer should be the preferred event occurring after the population of the 
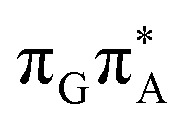
 of the T=TAG tetranucleotide. In contrast, direct photorelaxation of GAT=T from its 
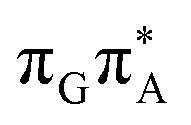
 state is again more efficient than for T=TAG, since the analogous S_1_(
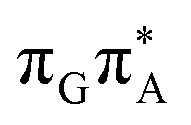
)/S_0_ state crossing lies 0.42 eV above and 0.25 eV below the G^+^˙A^−^˙ minimum located for the GA-*syn* and GA-*anti* conformers, respectively.

The above interpretation is further supported by the recent investigation of the photodynamics of the GA and AG dinucleotides with transient absorption spectroscopy, which showed that the yield of excited-state interbase charge transfer is higher by ∼75% for GA than for AG. Similarly the GA dinucleotide was reported to exhibit a longer lifetime of the CT state (170 ± 10 ps) than the AG dinucleotide (112 ± 12 ps).^55^ While in the case of these dinucleotides the trend is clearly opposite than for the damaged GAT=T and T=TAG tetranucleotides, dinucleotides are characterized by very different conformational spaces than longer oligomers owing to both purine bases being located at the termini of the mini-strands. Consequently, the results of Petropoulos *et al.*^55^ are consistent with the picture emerging from our QM/MM calculations, that is, that the population of DNA excited CT states is strongly affected by the interbase stacking pattern.

The second excited-state e^−^ transfer allows the tetranucleotide to reach the T=T^−^˙AG^+^˙ electronic configuration that enables direct photoreversal of the CPD. This CT event is associated with much stronger driving force for electron transfer as the T=T^−^˙AG^+^˙ S_1_ minimum lies 1.22 eV below the A^−^˙G^+^˙ minimum. Similarly as in the case of the GAT=T tetranucleotide as well as damaged trimers containing 2,6-diaminopurine, a very modest barrier (<0.1 eV) separates the final S_1_ CT minimum of T=TAG from the S_1_(
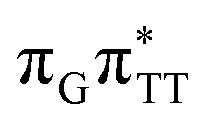
)/S_0_ state crossing responsible for CPD repair (see the PES presented in Fig. S10 in the ESI†). Beyond this barrier, the C5–C5 bond breaking process can occur spontaneously and the S_1_(
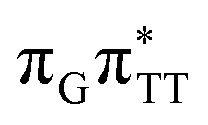
)/S_0_ conical intersection has a peaked topography (see Fig. S10 in the ESI†). This geometry of the T=T dimer in this MECP is characterized by the C5⋯C5 distance (between constituent T bases) equal to 2.55 Å, which means that this covalent bond of the CPD is completely broken at the point of conical intersection. The remaining C6–C6 bond of the CPD maintains its length of 1.56 Å at the S_1_/S_0_ state crossing, but can be subsequently broken in the hot electronic ground state, which completes the CPD self-repair process.^35,56,57^ In other words, CPD reversal is a stepwise process and as reported previously for enzymatic repair of thymine dimers, the barrier associated with C6–C6 bond breaking does not exceed 0.15 eV (3.1 kcal mol^−1^).^56,57^

We emphasize, that reaching this S_1_(
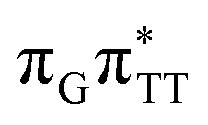
)/S_0_ state crossing does not ensure CPD self-repair, since the C5–C5 bond may still be reformed after non-radiative transition to the S_0_ state. Therefore, the experimentally measured self-repair quantum yield cannot be directly associated with the quantum yield of the SET process in T=TAG. However, the peaked topography of this state crossing indicates that C5–C5 bond rupture involves relatively high momentum, which should generally drive the C5 atoms of the T bases towards greater separation, which can be subsequently followed by C6–C6 bond breaking.^31,35^

## Conclusions

In summary, we demonstrated that the directionality of DNA sequences can substantially influence the efficiency of photoinduced electron transfer through the base stack. We present this based on the example of photoinduced self-repair of cyclobutane pyrimidine dimers in the GAT=T and T=TAG sequences, which is controlled by the sequential electron transfer mechanism.^35^ In particular, the T=TAG sequence is characterized by higher quantum yields of CPD self-repair when compared to the GAT=T tetramer (0.58% *vs.* 0.44%). Overall, the T=TAG tetranucleotide can repair up to 40% of the formed photodimers before reaching photostationary equilibrium when irradiated at 285 nm. In comparison, we managed to achieve only up to 33% of self-repair for the GAT=T sequences under equivalent conditions.

We ascribe this phenomenon to the differences in the conformational ensembles between the two tetranucleotides. Firstly, for the major conformers of the T=TAG tetramer (AG-*anti*), we observe much higher degree of stacking between the G and A bases than in the case of the GAT=T tetranucleotide. Better stacking overlap lowers the energy of 
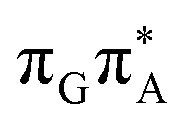
 CT state in the Franck–Condon region. This electronic CT state is responsible for the first electron transfer event in the CPD self-repair process. Secondly, the S_1_/S_0_ minimum-energy crossing points (MECPs) responsible for the direct photorelaxation of the intermediate 
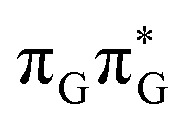
 LE and 
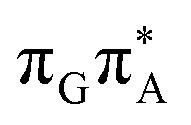
 CT states are practically energetically inaccessible in the T=TAG tetramer. As a result, excited-state electron transfer process is a much more competitive and favorable photorelaxation mechanism for T=TAG than in the case of the GAT=T tetranucleotide. Even though many more photorelaxation mechanisms are usually available in DNA strands, involving *e.g.* locally-excited nπ* or πσ* states, our calculations demonstrate that the well-stacked conformation of the T=TAG tetramer can effectively restrain some of these channels and promote interbase electron transfer.

We anticipate that these alternative photorelaxation mechanisms are the main reason for the modest self-repair quantum yields resulting from our measurements. Here, we were able to identify two other photorelaxation pathways of the intermediate 
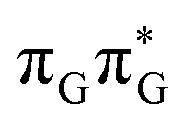
 LE and 
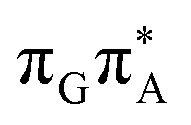
 CT electronic states with our static excited-state PES explorations. However, in a dynamic picture higher electronic states can easily interchange their order with the S_1_ state and lead to other S_1_/S_0_ state crossings, which are more challenging to grasp with the static QM/MM approach. These state crossings can potentially enable back-electron transfer which was observed for polyadenosine sequences.^58^ Furthermore, as discussed above, reaching the key S_1_(
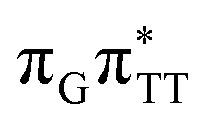
)/S_0_ state crossing which entails C5–C5 bond breaking may still be followed by CPD reformation. Nevertheless, the quantum yields are sufficiently high to enable the accumulation of high quantities of repaired material during continuous irradiation (∼33% for GAT=T and ∼40% for T=TAG).

Overall, we show that photoinduced electron transfer in DNA and the associated CPD self-repair process are strongly dependent on conformation and the availability of alternative (direct) photorelaxation channels of the intermediate states. This demonstrates that the efficiency of electron transfer cannot be simply predicted based on sequence. However, prior computational exploration of the conformational spaces of DNA sequences and their associated photochemical properties (including the energetics of CT states and S_1_/S_0_ state crossings) can offer valuable predictive capacity for the identification of DNA oligomers that can undergo efficient charge separation upon UV absorption.

## Data availability

Computational data, including geometries and results of QM/MM calculations can be found under: https://doi.org/10.6084/m9.figshare.24711825.

## Author contributions

Conceptualization: C. L. K. and R. S.; investigation and formal analysis: C. L. K., S. C., D. D., P. S. and R. S.; funding acquisition: D. D. S., J. W. S., J. S. and R. S.; resources: J. S. and J. W. S.; writing – original draft: R. S.; visualisation: C. L. K. and R. S.; writing – review and editing: C. L. K. and R. S.; supervision: D. D. S., C. L. K., J. W. S., J. S. and R. S.

## Conflicts of interest

There are no conflicts to declare.

## Supplementary Material

SC-015-D3SC04971J-s001

SC-015-D3SC04971J-s002
