# Supplementary material for: Photoinduced charge separation and DNA self-repair depend on sequence directionality and stacking pattern
Source: Chem Sci. 2023 Dec 28;15(6):2158–66. doi: 10.1039/d3sc04971j (PMC10848779; doi:10.1039/d3sc04971j)
Supplement: SC-015-D3SC04971J-s001 [file SC-015-D3SC04971J-s001.pdf]

Electronic Supplementary Material (ESI) for Chemical Science.
This journal is © The Royal Society of Chemistry 2023

Electronic Supplementary Information (ESI) for: Photoinduced charge separation and DNA self-repair depend on sequence directionality and stacking pattern

Corinna L. Kufner,*[a] Sarah Crucilla,[a,b] Dian Ding,[c,d] Petr Stadlbauer,[e,f] Jiří Šponer,[e,f] Jack W. Szostak,[g,h] Dimitar D. Sasselov,[a] and Rafał Szabla*[i]

December 1, 2023

SContents

1 Experimental Details **2**
1.1 Data Evaluation . . . 2
1.2 HPLC Analysis . . . 2
1.3 NMR Measurements . . . 2
1.4 Mass Spectrometry . . . 2

2 Computational Methods **3**
2.1 Molecular dynamics simulations . . . 3
2.2 Preparation of Initial Solvated Structures of the GAT=T tetranucleotide . . . 7
2.3 QM/MM Setups . . . 7
2.4 Geometry Optimizations . . . 8
2.5 Excited-State QM/MM Calculations . . . 9

3 Supplementary results and analyses **10**
3.1 UV-vis absorption of selected nucleosides and the emission profile of our light source . . . 10
3.2 Benchmark calculations for the sequential electron transfer mechanism . . . 10
3.3 Charge transfer character of the located S_1 minima . . . 11
3.4 Potential energy profile for CPD self-repair in the $\pi_G\pi^*_{TT}$ CT state . . . 11
3.5 Computational data availability . . . 12

4 References **12**

S1 Experimental Details

1.1 Data Evaluation

The sample preparation and the photometric setup have been described previously.[1] The absorption difference spectra in Figure 2B were acquired by subtraction of the unirradiated from the irradiated sample UV / Vis spectra at different doses. The absorbance around 360 nm was subtracted from all difference spectra. 10% reflection losses were assumed in the determination of the absorbed dose from the transmitted power. The absorbance difference datapoints at 266 nm were plotted as a function of the absorbed dose and the slopes were determined by linear fit to the datapoints 2. The calculation of the quantum yields from the linear fit has been described in detail.[1] Statistical errors were estimated from averaging over triplicate measurements. The systematic error was conservatively estimated to around 40%. The following errors were included in the estimate: 25% for spectral bandwidth of the LED, 20% for sample impurities, 15% for spectrophotometric measurements, 8% for pipetting inaccuracies, 5% for sample positioning inaccuracies during irradiation, 3% for Fresnel reflections, 2% for irradiation time intervals, 1% for beam focusing, and 1% for power detection.

1.2 HPLC Analysis

The analytical HPLC spectra were aquired on a reverse-phase system (Agilent 1100, ZORBAX Eclipse plus C18, 4.6 x 250mm, 5µm, 400 bar). Eluting buffers were: Buffer A: Acetonitrile (Sigma-Aldrich, 494445-2L) and buffer B: Triethylammonium bicarbonate (Life Technologies Corporation, 90114, 20mM in H2O). 5µL of sample was eluted over 38 minutes using a flow of 1 mL/min and a gradient of 5% to 20% of buffer B for each run at 25°C.

1.3 NMR Measurements

^{1}H spectra were acquired on a Varian Oxford AS-400 NMR spectrometer (400 MHz for ^{1}H) at 25°C. The chemical shifts are reported in parts per million (ppm) values on the δ scale. The samples were dissolved in D_2O and the ^{1}H NMR was referenced using internal standards: Sodium 2-(trimethylsilyl)-1-propanesulfonate-d6 (0 ppm at 25°C) for GATT and GAT=T, and triethylamine (TEA) (δ 1.13 (t, J = 7.3 Hz, ^{1}H), 3.05 (q, J = 7.3 Hz, ^{1}H) at 25°C) for TTAG and T=TAG. Occasionally, peaks corresponding to residual TEAB (3.19 and 1.27 ppm) and acetonitrile (2.08 ppm) from HPLC purification were observed. To diminish the presence of water in the ^{1}H spectra, the samples were dissolved in D_2O and lyophilized for multiple cycles.

1.4 Mass Spectrometry

HRMS was aquired on an Agilent 1200 HPLC coupled to an Agilent 6230 TOF mass spectrometer. The samples were separated by IP-RP-HPLC on a 100 mm × 1 mm Xbridge C18 column with 3.5 µm particle size (Water, Milford, MA) with (A) 200 mM 1,1,1,3,3,3-hexafluoro-2- propanol with 1 mM triethylamine, pH 7.0 and (B) methanol. The samples were eluted between 2.5% and 12% B over 16 min with a flow rate of 0.08 mL/min at 30°C. The acquired mass spectra (not shown) confirmed the expected masses of all oligonucleotides studied in this work: TTAG (1188 m/z), T=TAG (1188 m/z), GATT (1188 m/z), and GAT=T (1188 m/z).

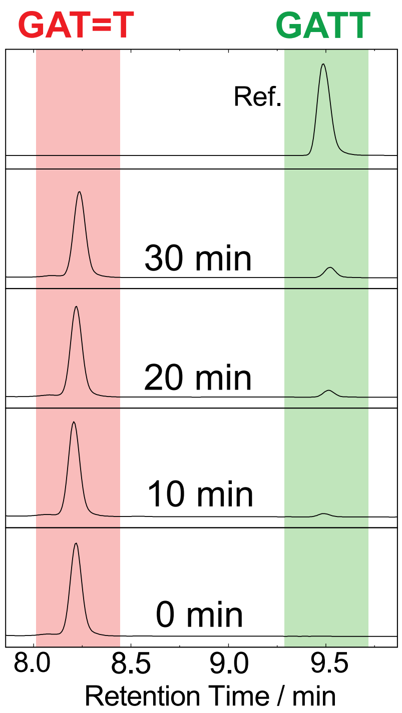

Fig. S 1 Analytical HPLC analysis of the self-repair of the sequence GAT=T to GATT. The damaged starting material GAT=T (8.3 min) gradually recovers to GATT (9.5 min) upon exposure to 285 nm irradiation. The chromatogram of the undamaged sequence GATT is shown at the top for comparison.

S2 Computational Methods

2.1 Molecular dynamics simulations

The bonding and non-bonding parameters for the cyclobutane T=T dimer were derived previously and we reused them for this study.[3] In this work, we are additionally, releasing these parameters in a separate compressed directory alongside the PDB structures from the clustering procedure (see below) as supplementary material to this work. The T=TAG tetranucleotide was solvated in a truncated octahedral box of water molecules with the distance between the oligonucleotide and border of the box being larger than 10 Å. We applied two different models of explicit water, namely the three-point SPC/E model[4] and the four-point OPC model[5] for comparison. At first, we neutralized the box with 3 K+ cations and added 0.15 M of KCl afterwards, Joung and Cheatham parameters for SPC/E and TIP4Pew,[6] respectively. For the oligonucleotide, we employed the parmOL15 force field containing reparamentrisations of the key dihedral angles,[7–10] which is based on the original DNA force field developed by Cornell and co-workers.[11] We emloyed the following equilibration protocol. Initial minimization of the system involved 500 steps of steepest descent and was followed by 500 steps of minimization with the conjugate gradient scheme. Both stages involved position restraints on the solute atoms set to 25 kcal·mol^{-1}. The system was then heated from 0 to 300K in a simulation lasting 100 ps, with the same position restraints of 25 kcal·mol^{-1} imposed on the DNA fragment. We performed the heatingh at constant volume. Afterwards, we minimized the system with 5 kcal·mol^{-1} restraints in the oligonucleotide again using 500 steps of steepest descent followed by 500 steps of the conjugate gradient optimization approach. We next performed an equilibration for 50 ps at constant temperature of 300 K and constant pressure of 1 bar. This was followed by an analogous series

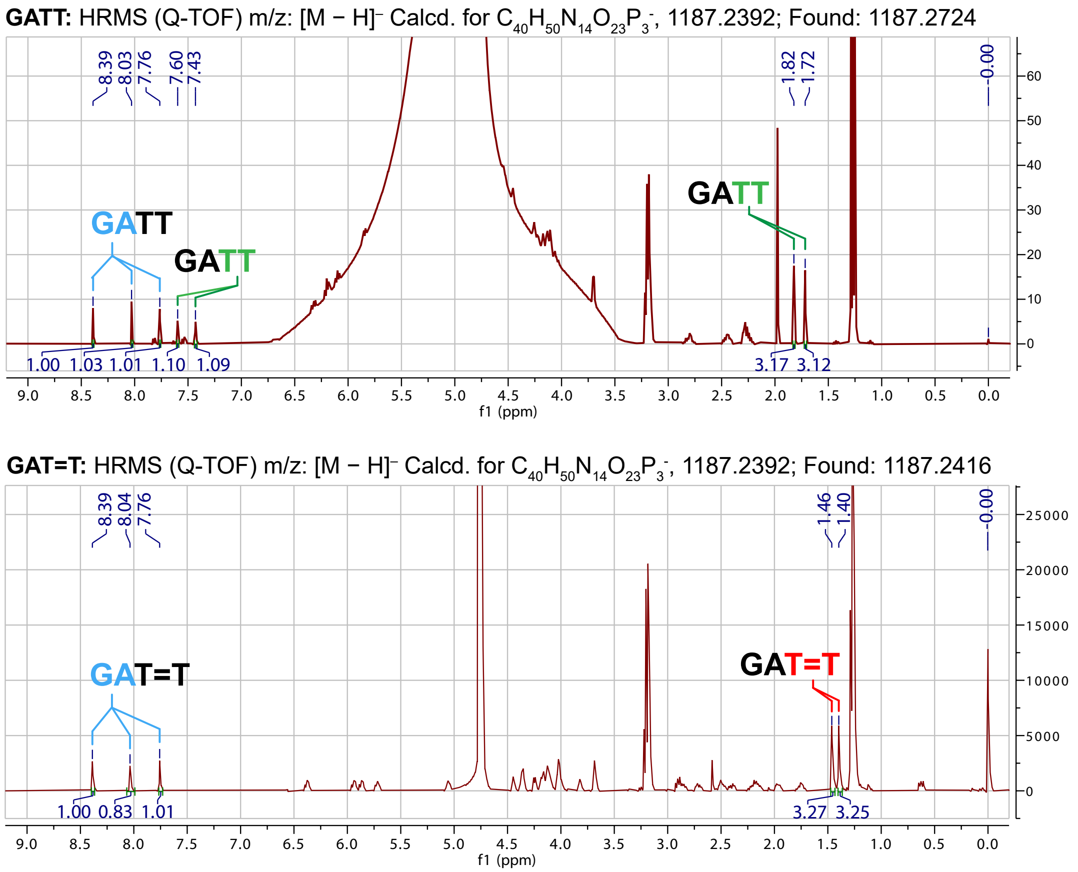

Fig. S 2 HRMS and NMR spectra of the undamaged sequence GATT (top) and the damaged sequence GAT=T (bottom) in D_2O. In very good agreement with the literature[2], the chemical shifts of the methyl group on thymine (green) change from 1.82 ppm and 1.72 ppm to 1.46 pmm and 1.40 ppm upon dimer formation (red). Also, the upfield shift of the thymine alkenyl hydrogen peaks at 7.60 ppm and 7.43 ppm are indicative of dimer formation. Identification of the exact position of the CPD hydrogen peaks is difficult as they appear in the same region as the water peak and sugar back bone hydrogen peaks. The chemical shifts and integration of the proton peaks on adenine and guanine (blue) (8.39 ppm, 8.03 ppm, 7.76 ppm) are indicated.

TTAG: HRMS (Q-TOF) m/z: [M − H]⁻ Calcd. for $C_{40}H_{50}N_{14}O_{23}P_3^-$, 1187.2392; Found: 1187.2477

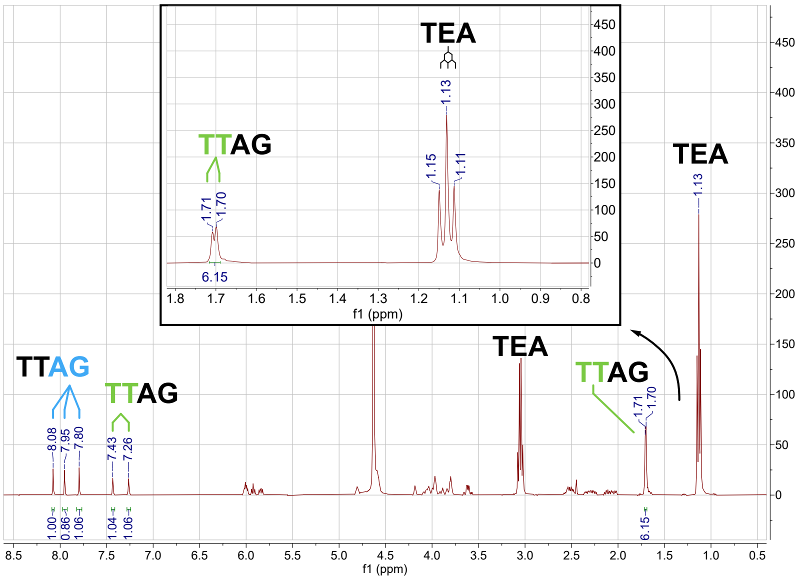

T=TAG: HRMS (Q-TOF) m/z: [M − H]⁻ Calcd. for $C_{40}H_{50}N_{14}O_{23}P_3^-$, 1187.2392; Found: 1187.2459

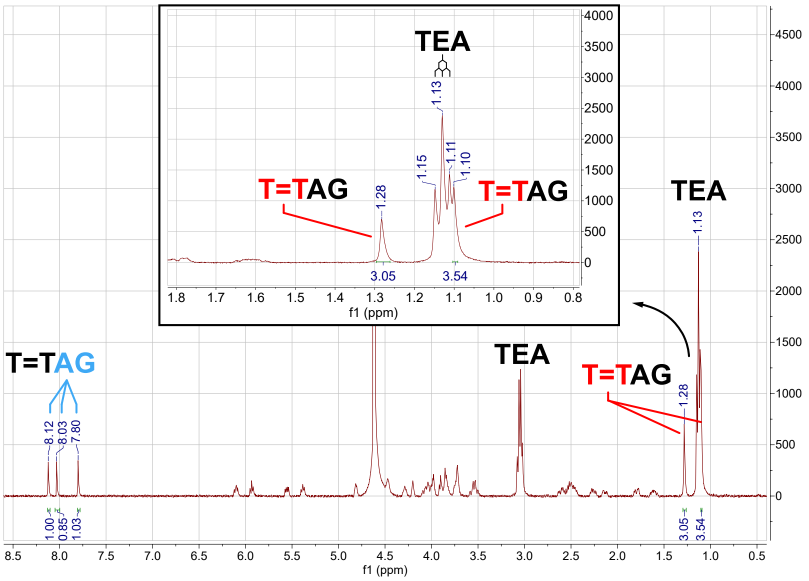

Fig. S 3 HRMS and NMR spectra of the undamaged sequence TTAG (top) and the damaged sequence T=TAG (bottom) in D_2O. The chemical shifts of the methyl group on thymine (green) change from 1.71 ppm and 1.70 ppm to 1.28 ppm and 1.10 ppm upon dimer formation (red). The upfield shift of the thymine alkenyl hydrogen peaks at 7.43 ppm and 7.26 ppm also indicate dimer formation. The CPD hydrogen peaks appear in the same region as the water and sugar back bone hydrogen peaks. Therefore, identifying the exact position is difficult. The chemical shifts and integration of the proton peaks on adenine and guanine (blue) (8.12 ppm, 8.03 ppm, 7.80 ppm) are indicated.

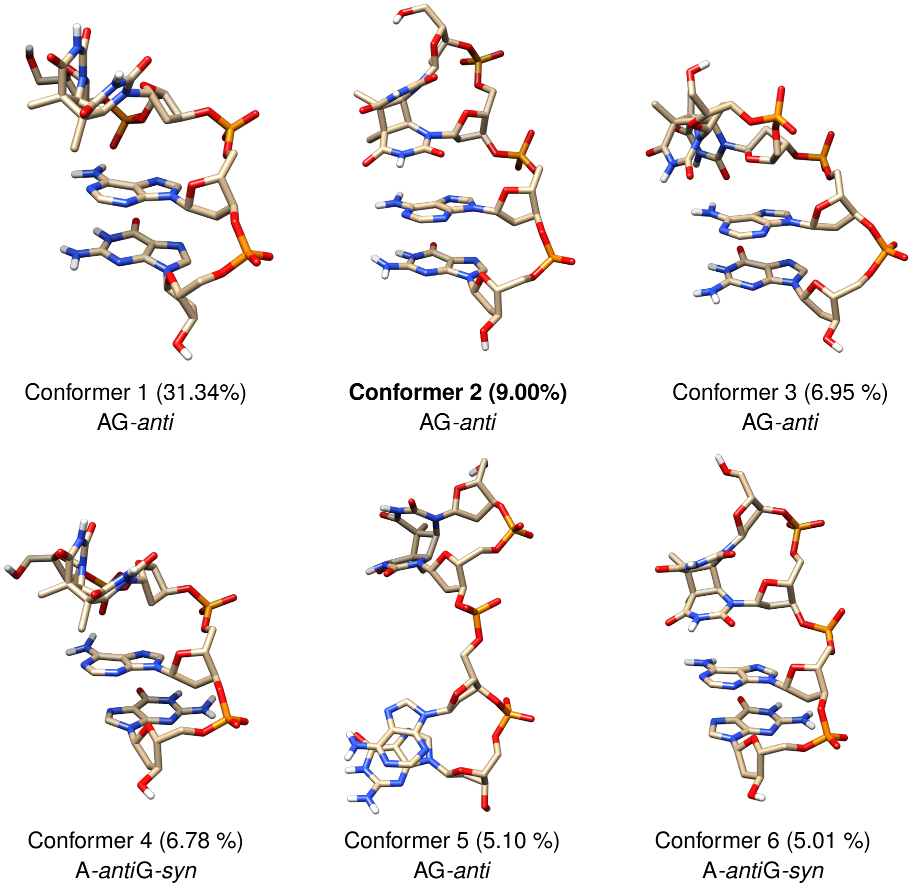

Fig. S 4 Clusters with populations exceeding 5.0% obtained from the clustering procedure for the 10μ MD simulation performed for the T=TAG tetranucleotide solvated in a periodic (truncated octahedron) box of explicit SPC/E water molecules. Primary set of QM/MM calculations was performed for Cluster 2.

of consecutive minimization and equilibration runs involving decreasing position restraints on the DNA atoms of 4, 3, 2, 1 and 0.5 kcal·mol^{-1}. Initial velocities for each simulation were taken from the preceding equilibration steps. This series was followed by an unrestrained molecular dynamics simulation with the length of 50 ps. The coupling constants necessary to control the temperature and pressure were set to 0.2 ps during the equilibration phase, and to 5 ps during the final phase of unrestrained molecular dynamics. Next, we performed the producrtion runs with the length of 10 μs for each solvent model. During the production phase, we applied the hydrogen mass repartitioning scheme[12] as well as the SHAKE[13] and SETTLE[14] algorithms, which allowed us to set the time step to 4 fs. We used the Berendsen weak-coupling thermostat and barostat[15] to maintain the temperature and pressure at 300 K and 1 bar, respectively. We applied the Particle Mesh Ewald method (PME) for the electrostatic interactions. The cutoff for non-bonding interactions was set to 9 Å. The calculations were performed using the CUDA accelerated pmemd module of AMBER 16.[16]

After the production runs, we performed clustering of the trajectories to find the most representative and

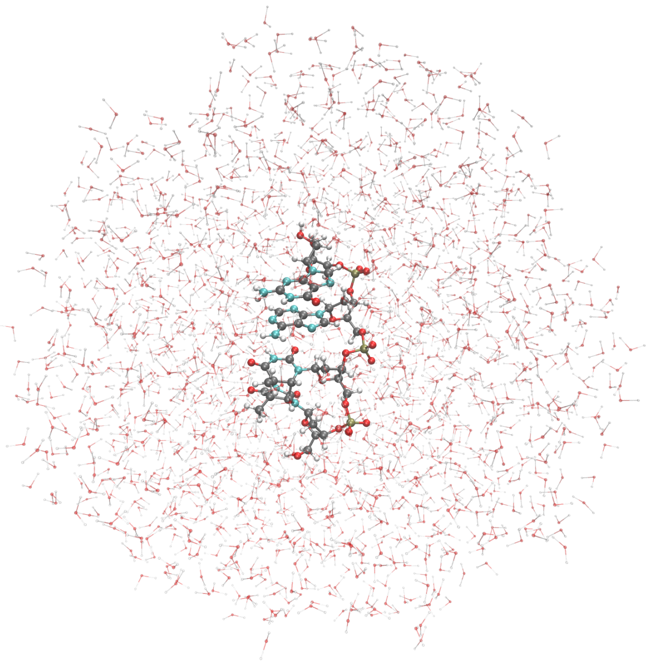

Fig. S 5 The T=TAG tetranucleotide solvated with the sphere of explicit water molecules which was used in the QM/MM simulations.

relevant conformers. For this purpose, we applied a modification of the algorithm proposed by Rodriguez and Laio, and used eRMSD as the clustering metric.[17,18] This metric is specific for nucleic acids and it allows to determine the similarity of two structures based on relative orientation of their nucleobases and is a suitable tool for distinguishing nucleotides *syn-* and *anti-* orientations or conformations with different degree of stacking. For the sake of further analysis, we selected clusters with a population exceeding 1%.

2.2 Preparation of Initial Solvated Structures of the GAT=T tetranucleotide

Following the analysis and clustering of the simulated trajectories for the T=TAG tetranucleotide, we selected the most representative stacked conformations for subsequent QM/MM simulations. These conformations are characterized by structural characteristics typical also for longer oligonucleotides. Consequently many of our qualitative findings and outcomes of excited-state calculations are applicable to extended DNA fragments as well. Notably, the stacked GA-*anti* arragment of the purine bases in the the T=TAG tetramer is their most prevalent conformation in MD simulations utilizing the SPC/E and OPC solvent models. While the unstacked structures have a substantial contribution to the overall dynamics of T=TAG, the fully stacked form selected for further simulations is also among the highest populated structures for this tetranucleotide. The tetranucleotides were immersed in a sphere of explicit water molecules with a radius of 24 Å, and the solvent was equilibrated for 10 ps while restraining the solute (tetranucleotide). The resulting geometries were subsequently used for ground-state geometry optimizations.

2.3 QM/MM Setups

We employed two different QM/MM partinioning setups: one including the entire trinucleotide in the QM region (QM_{DNA}/MM setup), while the other one included only the nucleobases treated at the QM level

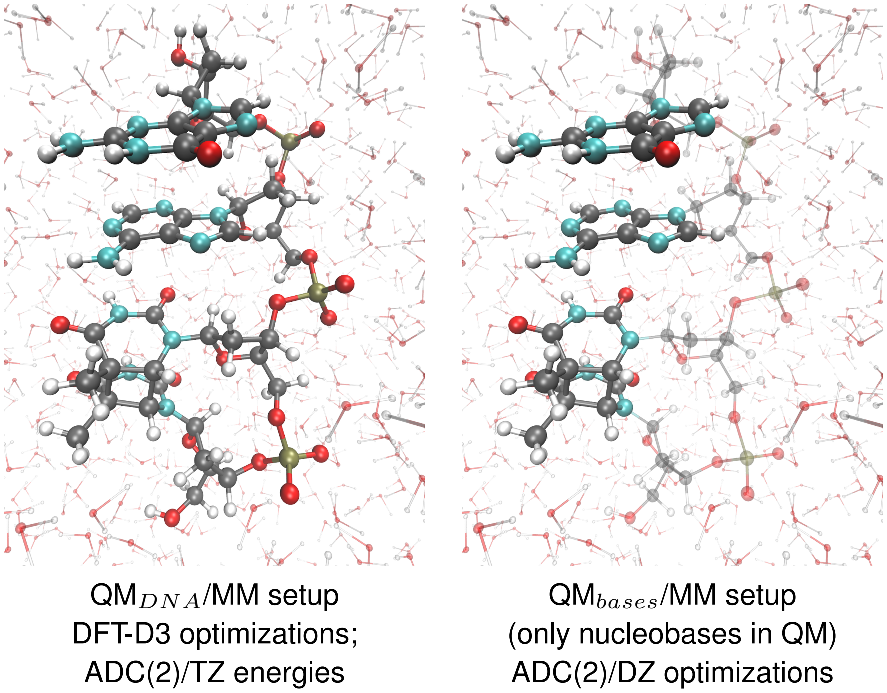

Fig. S 6 The two QM/MM setups considered in this work. Solid models show the QM region, while the partly transparent models correspond to the MM region.

(QM$_{bases}$/MM setup), as depicted in Figure 6. The remaining components of the system (water molecules and sugar-phosphate backbone for the latter setup) were described using the same force-field suite as in the MD simulations, including the SPC/E force field for water. The calculations within the QM$_{bases}$/MM setup were performed using the link hydrogen atom scheme with a MM charge of 0 on the link H atom. The QM$_{bases}$ and MM regions were separated by bisecting the the N-glycosidic bonds. Electrostatic embedding was adopted for all QM/MM calculations, facilitated by our in-house modification of the QM/MM interface within the AMBER suite of programs,[19] which enables QM calculations with the TURBOMOLE 7.3 program.

2.4 Geometry Optimizations

We optimized the minimum-energy geometries of the selected conformers of the damaged T=TAG tetranucleotide using the cost-effective composite PBEh-3c hybrid DFT method and the QM$_{DNA}$/MM setup for electronic-structure calculations.[20] The PBEh-3c functional was demonstrated to yield accurate structures and relative energetics for nucleic acid oligomers and their conformers.[21] Initial optimizations of the minimum-energy geometries in the electronic ground state were conducted for the entire solvated system with the AMBER limited-memory BFGS algorithm. A more rigorous optimization of an internal spherical region (within a radius of 12.5 Å from the central atom of each conformer) was performed using the open-source external geometry optimizer XOPT,[22] employing the approximate normal coordinates and rational function schemes.[23] The outer region of the sphere remained fixed during this procedure. We applied this approach to all ground-state and excited-state geometry optimizations.

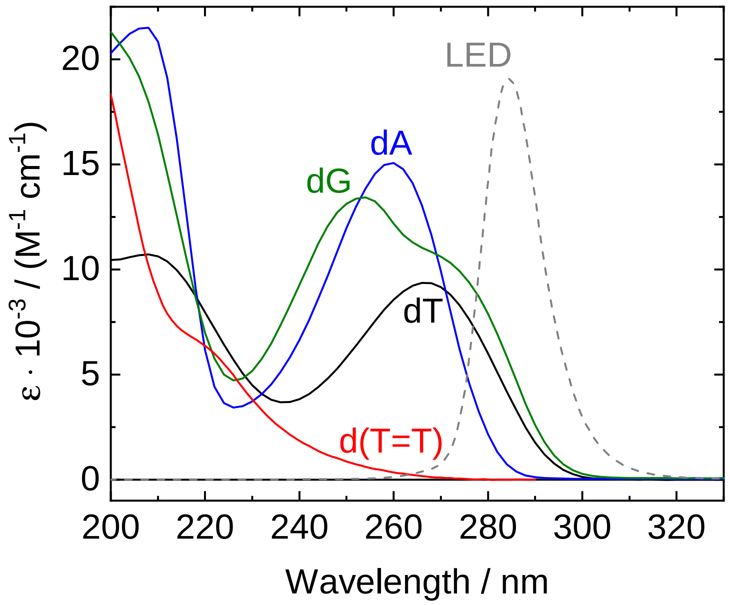

Fig. S 7 Molar decadic absorption coefficients /epsilon of the d(T=T) CPD lesion (red) and the undamaged mononucleotide dT (black), dG (green), and dA (blue), following the results and discussion presented by Schreier *et al.*[24] The emission spectrum of the LED centered around 285 nm, which was used for irradiation, is shown as grey dashed line for comparison.

2.5 Excited-State QM/MM Calculations

We used the algebraic diagrammatic construction to second order [ADC(2)] method[25–27] for all excited-state energy and gradient calculations performed within the QM/MM framework. The corresponding ground-state energies and gradients were obtained at the MP2 level. All calculations required excited-state energy gradient were performed with the def2-SVP basis set, which yielded consistent results with the larger TZVP basis set in our previous investigations of the DNA self-repair mechanism.[3] In addition, in section 3.2, we are presenting benchmark QM/MM calculations with the larger TZVP basis set and larger QM region for the sequential electron transfer mechanism. Vertical excitation energies presented in Table 1 of the main article were computed using the QM_{DNA}/MM setup based on the minimum-energy ground-state geometries optimized with the PBEh-3c method.[20] Optimizations of various minima on the S_1 potential energy surface and S_1/S_0 minimum-energy crossing points (MECPs) were conducted using the QM_{bases}/MM setup. The characters of electronically excited states were assigned through the analysis of molecular orbitals and the key configurations contributing to a given electronic excitation. The charge transfer character was determined by means of the electron-hole population analysis using the TheoDORE 1.5.1 package.[28,29] MECP optimizations were conducted with the approach developed by Levine, Coe, and Martínez,[30] which allows to identify highly representative geometries of S_1/S_0 conical intersections without the calculation of nonadiabatic couplings. Although the single-reference ADC(2)/MP2 approach is not capable of correctly reproducing the two coordinates responsible for lifting the degeneracy at conical intersections, it was successfully used to locate minimum-energy state crossings in various organic aromatic chromophores and it was shown to accurately describe the associated $S_1(\pi\pi^*)/S_0$ MECP geometries, including correct PE surface topographies up to the state crossing.[31,32] The energy values of key stationary points (S_0 and

S_1 minima, MECPs, and vertical excitation energies) were employed to construct illustrative excited-state potential-energy diagram in Figure 6 in the main article. Barrierless profiles between the Franck-Condon region of each and the S_1 minima were confirmed by excited-state geometry optimizations.

S3 Supplementary results and analyses

3.1 UV-vis absorption of selected nucleosides and the emission profile of our light source

In order to evaluate the contributions of different nucleobases to the overall absorption of the T=TAG tetranucleotides, we plotted the absorption spectra of canonical and damaged nucleobases considered in this work against the emission spectrum of our light source (LED, seef Fig. 7). These spectra clearly demonstrate that guanine the primary absorber of UV light in the case of the T=TAG tetramer in our experimental set-up. Therefore, the description of the mechanism, focuses on initial population of electronically excited states of guanine.

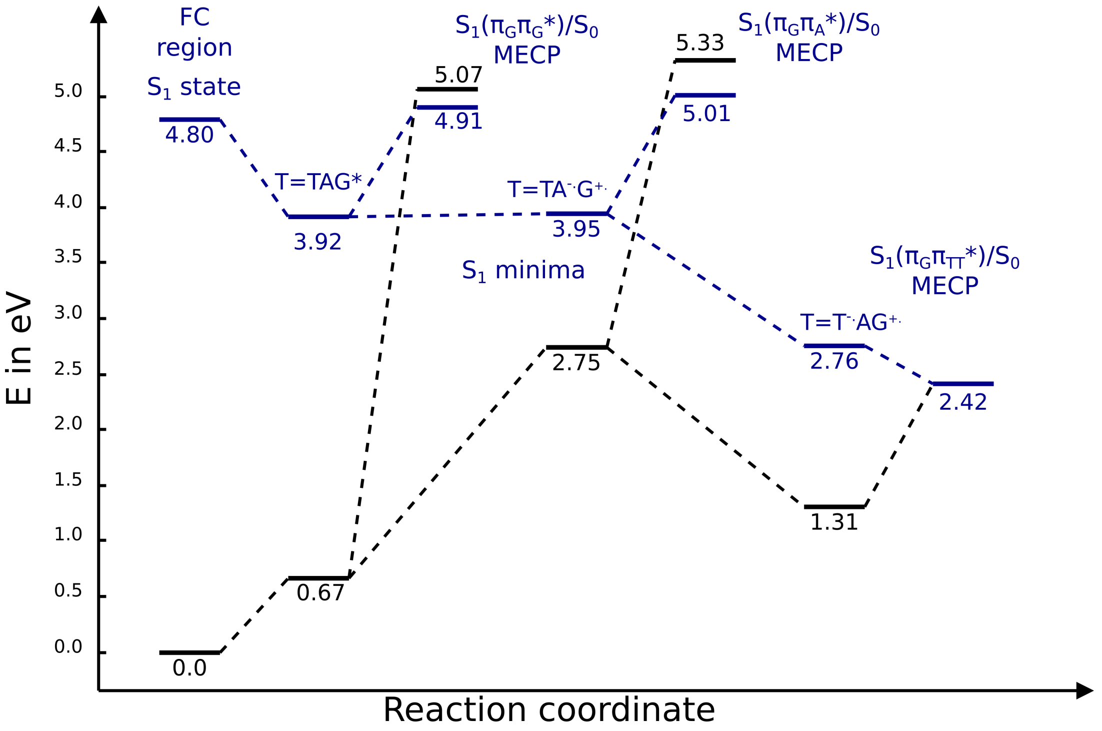

Fig. S 8 Benchmark calculations for the sequential electron transfer mechanism presenting the associated key minima on the S_1 excited-state potential energy surface and the considered minimum-energy crossing points (MECPs). The energies were calculated with the QM_{DNA}/MM setup with the ADC(2) method and a larger TZVP basis set. We used the same structures as in Fig. 6 in the main article, which were optimized with the QM_{bases}/MM setup and the ADC(2)/def2-SVP method.

3.2 Benchmark calculations for the sequential electron transfer mechanism

In order to validate the energies obtained with the ADC(2)/def2-SVP method and the QM_{bases}/MM setup for the sequential electron transfer mechanism (Fig. 6 in the main article), we performed additional calculations using a larger TZVP basis set and including the whole T=TAG tetranucleotide in the QM region.

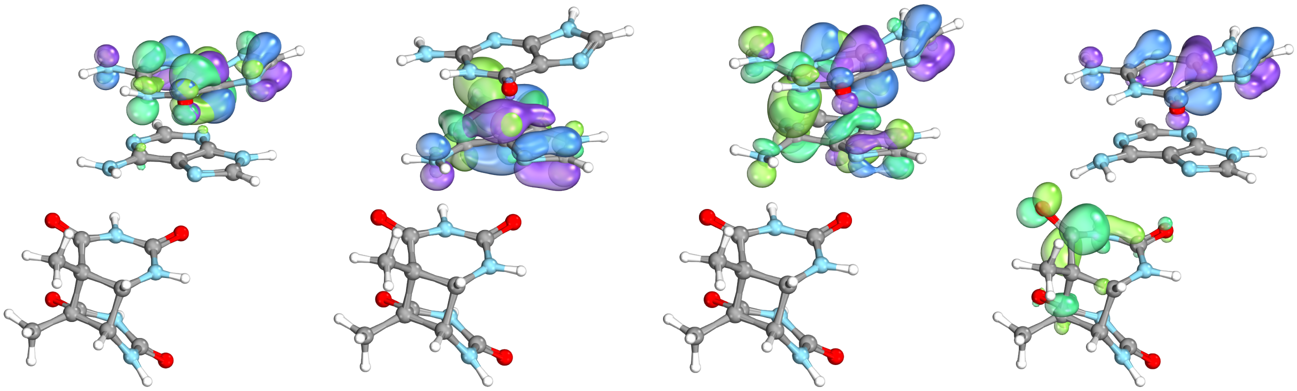

Fig. S 9 Molecular orbitals characterizing the character of the first excited state in different excited-state minima discussed in the main article. Blue and purple color corresponds to the the orbital from which the electron was excited (HOMO in the S_1 minima) whereas green color denotes the molecular orbitals which accept the excited electron (LUMO in the S_1 minima).

These results are presented in Fig. 8. Overall, both data-sets are highly consistent and the biggest discrepancy can be observed for the $S_1(\pi_G\pi_G^*)/S_0$ and $S_1(\pi_G\pi_A^*)/S_0$ state crossings. Both of these minimum-energy crossing points were optimized with the QM$_{bases}$/MM setup and recalculation of the energies with a larger basis set for the whole tetranucleotide resulted in energy splitting between the S_1 and S_0 states. The negative excitation energies indicate the these state crossings might be attained with slightly smaller structural displacements from the S_1 minimum energy geometries, however, the sloped topography of these crossings remains unchanged. In fact, the small ΔE_{S1-S0} (below 0.35 eV) for these structures, shows that they represent the corresponding conical intersections well in qualitative terms.

3.3 Charge transfer character of the located S_1 minima

In order to further confirm the charge-transfer (CT) character of the excited-state (S_1) minima involved in the proposed sequential electron transfer mechanism for the T=TAG tetranucleotide, we performed wave function particle-hole analysis with the TheoDore package.[28,29] The molecular orbitals best describing the involved electronic transitions are presented in Fig. 9. The excited-state minima found for the locally excited A and G bases do not exhibit any CT character, which is evident from the molecular orbitals (Fig. 9) and the particle-hole analysis indicating no charge transfer to neighbouring bases. The T=TA$^{-\cdot}$G$^{+\cdot}$ S_1 minimum involves a total transfer of 0.26 of an electron from the G base to the A base. The final T=T$^{-\cdot}$AG$^{+\cdot}$ S_1 minimum involves a transfer of 0.96 of an electron from the G base to the T=T dimer. This demonstrates that the hole is localized on the G base and the CPD repair process requires nearly a full electron to be transferred to lesion.

3.4 Potential energy profile for CPD self-repair in the $\pi_G\pi_{TT}^*$ CT state

Fig. 10 demonstrates the final stage of the CPD self-repair mechanism as presented in Fig. 6 in the main article. The presented PE profile was obtained as a relaxed scan performed on the S_1 hypersurface by fixing the C5–C5 distance at each consecutive step. For C5–C5 distances below 1.8 Å, the energy of the S_1 slightly increases, which is indicative of a very shallow minimum. After overcoming the barrier of <0.1 eV the C5–C5 bond breaking process is essentially barrierless and the S_1/S_0 is attained for the

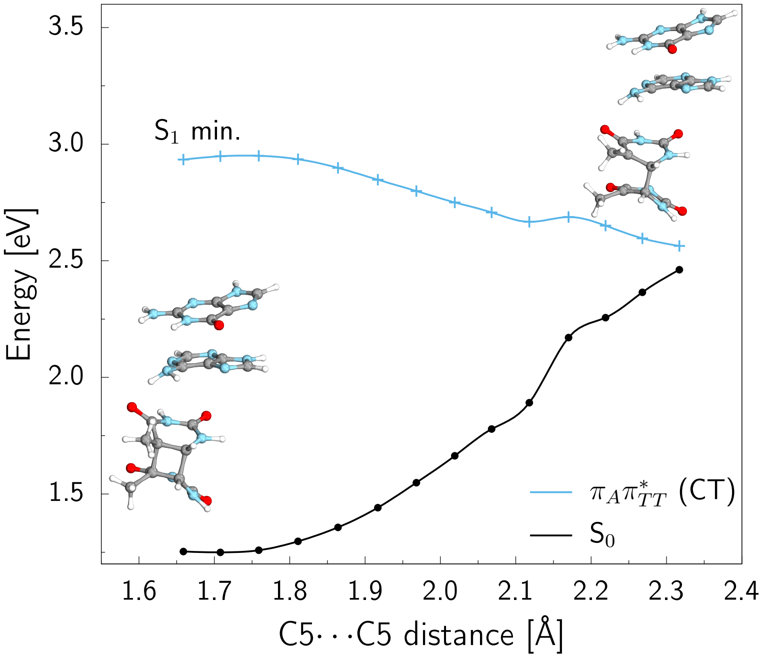

Fig. S 10 Potential energy (PE) profile for the C5–C5 bond breaking in the $\pi_G\pi^*_{TT}$ CT state. The profile was obtained as a relaxed scan with fixed C5–C5 bond length, while other coordinates were optimized on the S_1 excited-state PE surface at the ADC(2)/def2-SVP level, using the QM_{bases}/MM setup. This PE profile corresponds to the last stage of the CPD self-repair mechanism presented in Fig. 6 in the main article.

distance slightly exceeding 2.3 Å. The C5–C5 bond distance is very similar to the one calculated for the DT=T trinucleotide containing the alternative nucleobase 2,6-diaminopurine[33] and the canonical damaged GAT=T tetranucleotide[3]. In both cases, the S_1/S_0 minimum energy state crossing was located slightly above 1.82 Å. In addition the very shallow minimum of the $\pi_G\pi^*_{TT}$ in found for the T=TAG tetramer is consistent with the findings for the GAT=T tetranucleotide of opposite directionality. Consequently, the efficiency of the final CPD repair step is not affected by the directionality of the sequence (T=TAG vs. GAT=T) and the differences in quantum yields can be ascribed to the electron transfer quantum yields as discussed in the main article.

3.5 Computational data availability

The computational data generated for this project are freely available can be found on figshare.com under the DOI: 10.6084/m9.figshare.24711825. The data include PDB and XYZ structures, results of QM/MM calculations and results of clustering of MD trajectories.

S4 References

1 S. J. Crucilla, D. Ding, G. G. Lozano, J. W. Szostak, D. D. Sasselov and C. L. Kufner, *Chem. Comm.*, 2023, **59**, 13603–13606.
2 T. M. Koning, J. J. van Soest and R. Kaptein, *European journal of biochemistry*, 1991, **195**, 29–40.
3 R. Szabla, H. Kruse, P. Stadlbauer, J. Šponer and A. L. Sobolewski, *Chem. Sci.*, 2018, **9**, 3131–3140.
4 H. J. C. Berendsen, J. R. Grigera and T. P. Straatsma, *J. Phys. Chem.*, 1987, **91**, 6269–6271.

5 S. Izadi, R. Anandakrishnan and A. V. Onufriev, *J. Phys. Chem. Lett.*, 2014, **5**, 3863–3871.
6 I. S. Joung and T. E. I. Cheatham, *J. Phys. Chem. B*, 2008, **112**, 9020–9041.
7 M. Zgarbová, J. Šponer, M. Otyepka, T. E. Cheatham, R. Galindo-Murillo and P. Jurečka, *J. Chem. Theor. Comput.*, 2015, **11**, 5723–5736.
8 M. Zgarbová, F. J. Luque, J. Šponer, T. E. Cheatham, M. Otyepka and P. Jurečka, *J. Chem. Theor. Comput.*, 2013, **9**, 2339–2354.
9 A. Pérez, I. Marchán, D. Svozil, J. Sponer, T. E. I. Cheatham, C. A. Laughton and M. Orozco, *Biophys. J.*, 2007, **92**, 3817–3829.
10 M. Krepl, M. Zgarbová, P. Stadlbauer, M. Otyepka, P. Banáš, J. Koča, T. E. Cheatham, P. Jurečka and J. Šponer, *J. Chem. Theor. Comput.*, 2012, **8**, 2506–2520.
11 W. D. Cornell, P. Cieplak, C. I. Bayly, I. R. Gould, K. M. Merz, D. M. Ferguson, D. C. Spellmeyer, T. Fox, J. W. Caldwell and P. A. Kollman, *J. Am. Chem. Soc.*, 1995, **117**, 5179–5197.
12 C. W. Hopkins, S. Le Grand, R. C. Walker and A. E. Roitberg, *J. Chem. Theory Comput.*, 2015, **11**, 1864–1874.
13 J.-P. Ryckaert, G. Ciccotti and H. J. C. Berendsen, *J. Comput. Phys.*, 1977, **23**, 327–341.
14 S. Miyamoto and P. A. Kollman, *J. Comput. Chem.*, 1992, **13**, 952–962.
15 H. J. C. Berendsen, J. P. M. Postma, W. F. van Gunsteren, A. DiNola and J. R. Haak, *J. Chem. Phys.*, 1984, **81**, 3684–3690.
16 D. Case, J. Berryman, R. Betz, D. Cerutti, T. Cheatham III, T. Darden, R. Duke, T. Giese, H. Gohlke, A. Goetz, N. Homeyer, S. Izadi, P. Janowski, J. Kaus, A. Kovalenko, T. Lee, S. Legrand, P. Li, T. Luchko, R. Luo, B. Madej, K. Merz, G. Monard, P. Needham, H. Nguyen, H. Nguyen, I. Omelyan, A. Onufriev, D. Roe, A. Roitberg, R. Salomon-Ferrer, C. Simmerling, W. Smith, J. Swails, R. Walker, J. Wang, R. Wolf, X. Wu, D. York and P. Kollman, *AMBER 16*, 2016.
17 S. Bottaro, F. DiPalma and G. Bussi, *Nucleic Acids Res.*, 2014, **42**, 13306–13314.
18 A. Rodriguez and A. Laio, *Science*, 2014, **344**, 1492–1496.
19 A. W. Götz, M. A. Clark and R. C. Walker, *J. Comput. Chem.*, 2014, **35**, 95–108.
20 S. Grimme, J. G. Brandenburg, C. Bannwarth and A. Hansen, *J. Chem. Phys.*, 2015, **143**, 054107.
21 R. Szabla, M. Havrila, H. Kruse and J. Šponer, *J. Phys. Chem. B*, 2016, **120**, 10635–10648.
22 H. Kruse, *https://github.com/hokru/xopt; local development version.*, 2016, Institute of Biophysics, Brno.
23 F. Eckert, P. Pulay and H.-J. Werner, *J. Comput. Chem.*, 1997, **18**, 1473–1483.
24 W. J. Schreier, P. Gilch and W. Zinth, *Annual review of physical chemistry*, 2015, **66**, 497–519.
25 C. Hättig, *Advances in Quantum Chemistry*, Academic Press, 2005, vol. 50, pp. 37–60.
26 A. Dreuw and M. Wormit, *Wiley Interdiscip. Rev. Comput. Mol. Sci.*, 2015, **5**, 82–95.
27 A. B. Trofimov and J. Schirmer, *J. Phys. B: At. Mol. Opt. Phys.*, 1995, **28**, 2299.
28 F. Plasser, M. Wormit and A. Dreuw, *J. Chem. Phys.*, 2014, **141**, 024106.
29 F. Plasser, S. A. Bäppler, M. Wormit and A. Dreuw, *J. Chem. Phys.*, 2014, **141**, 024107.
30 B. G. Levine, J. D. Coe and T. J. Martínez, *J. Phys. Chem. B*, 2008, **112**, 405–413.
31 R. Szabla, R. W. Góra and J. Šponer, *Phys. Chem. Chem. Phys.*, 2016, **18**, 20208–20218.
32 D. Tuna, D. Lefrancois, Wolański, S. Gozem, I. Schapiro, T. Andruniów, A. Dreuw and M. Olivucci, *J. Chem. Theory Comput.*, 2015, **11**, 5758–5781.
33 R. Szabla, M. Zdrowowicz, P. Spisz, N. J. Green, P. Stadlbauer, H. Kruse, J. Šponer and J. Rak, *Nat.*

Commun., 2021, **12**, 3018.
