# Supplementary figures and images for: Photoinduced charge separation and DNA self-repair depend on sequence directionality and stacking pattern
Source: Chem Sci. 2023 Dec 28;15(6):2158–66. doi: 10.1039/d3sc04971j (PMC10848779; doi:10.1039/d3sc04971j)

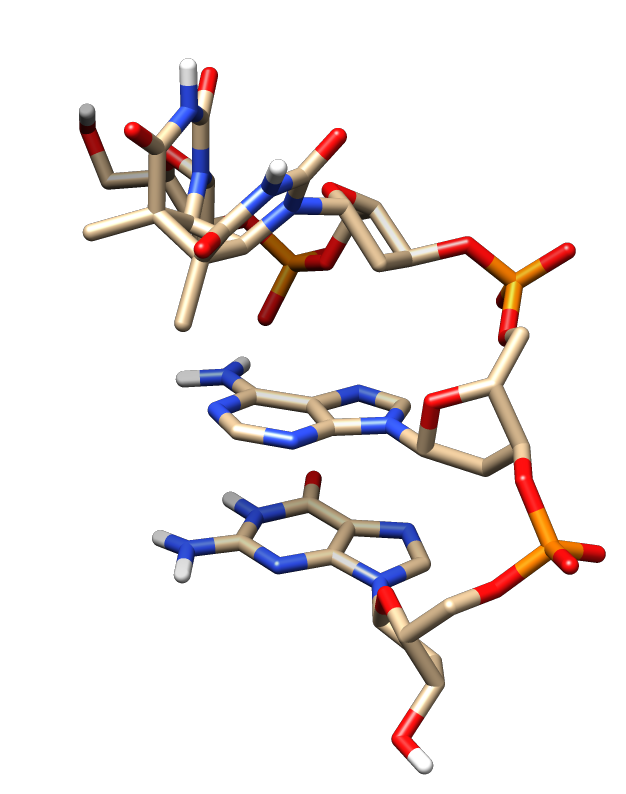

Supplement: SC-015-D3SC04971J-s002 [file SC-015-D3SC04971J-s002.zip › structures/clustering-based-on-MD/ttag_spce/cluster1.png]

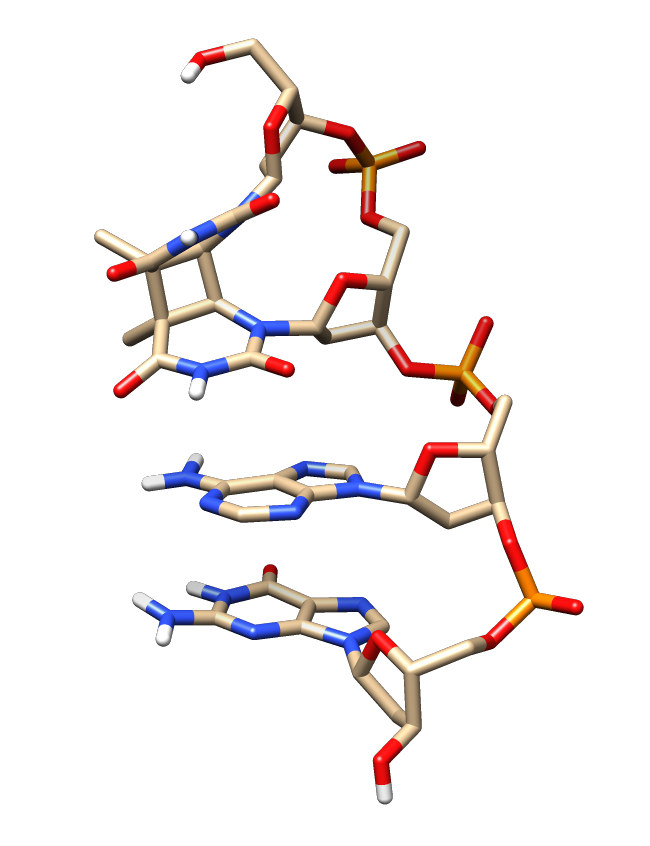

Supplement: SC-015-D3SC04971J-s002 [file SC-015-D3SC04971J-s002.zip › structures/clustering-based-on-MD/ttag_spce/cluster10.png]

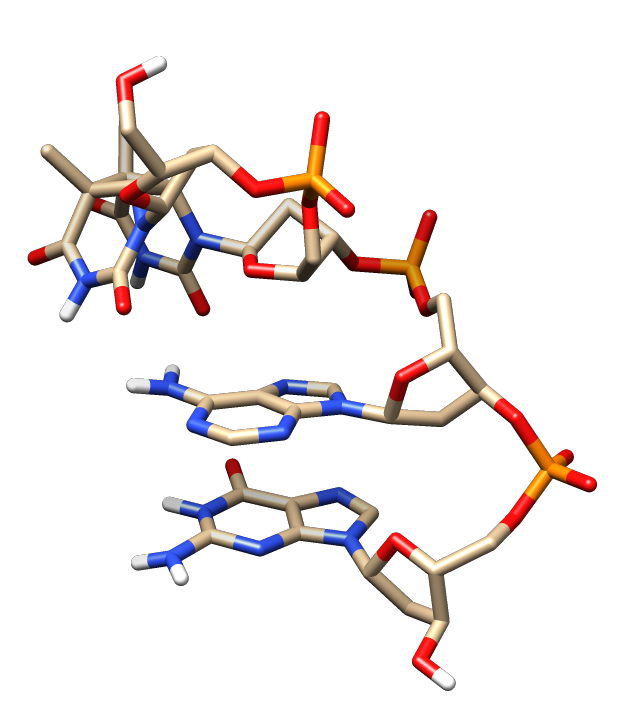

Supplement: SC-015-D3SC04971J-s002 [file SC-015-D3SC04971J-s002.zip › structures/clustering-based-on-MD/ttag_spce/cluster11.png]

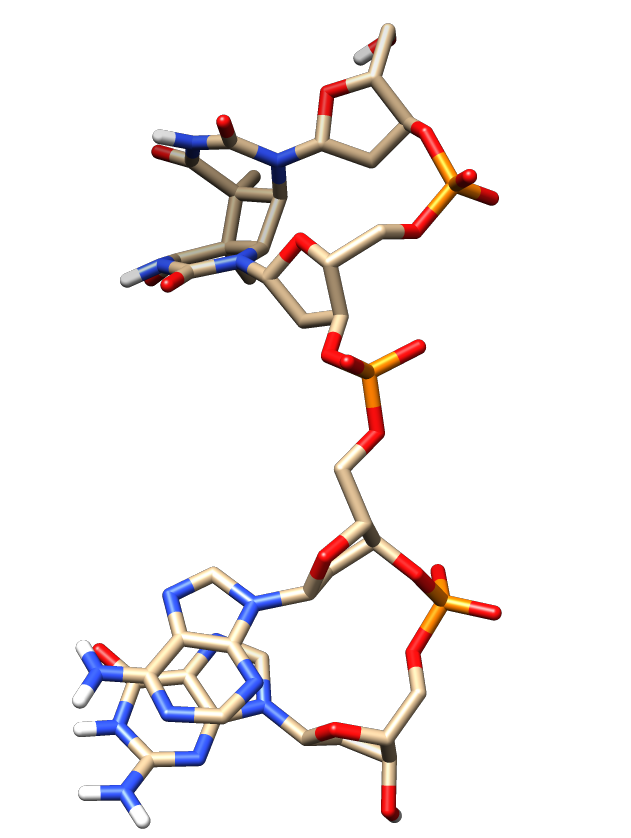

Supplement: SC-015-D3SC04971J-s002 [file SC-015-D3SC04971J-s002.zip › structures/clustering-based-on-MD/ttag_spce/cluster3.png]

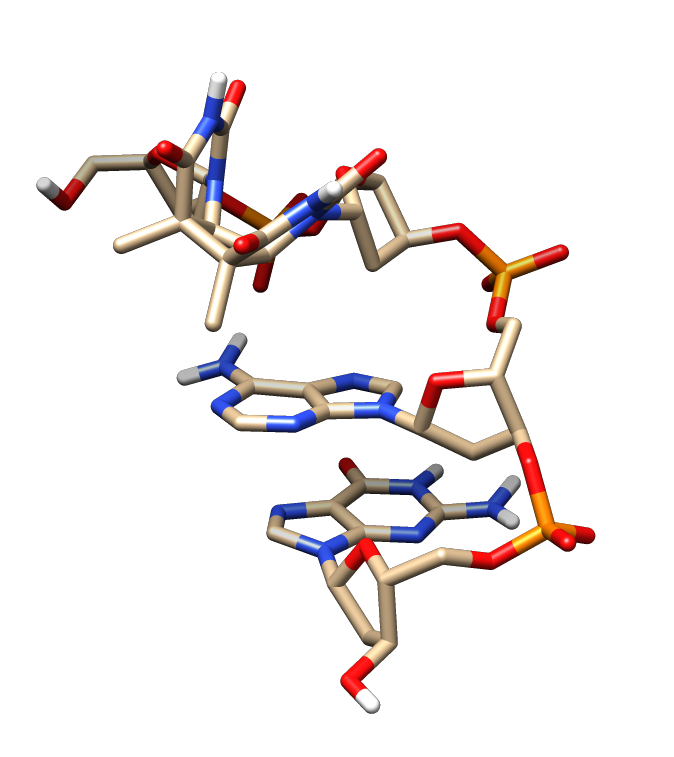

Supplement: SC-015-D3SC04971J-s002 [file SC-015-D3SC04971J-s002.zip › structures/clustering-based-on-MD/ttag_spce/cluster6.png]

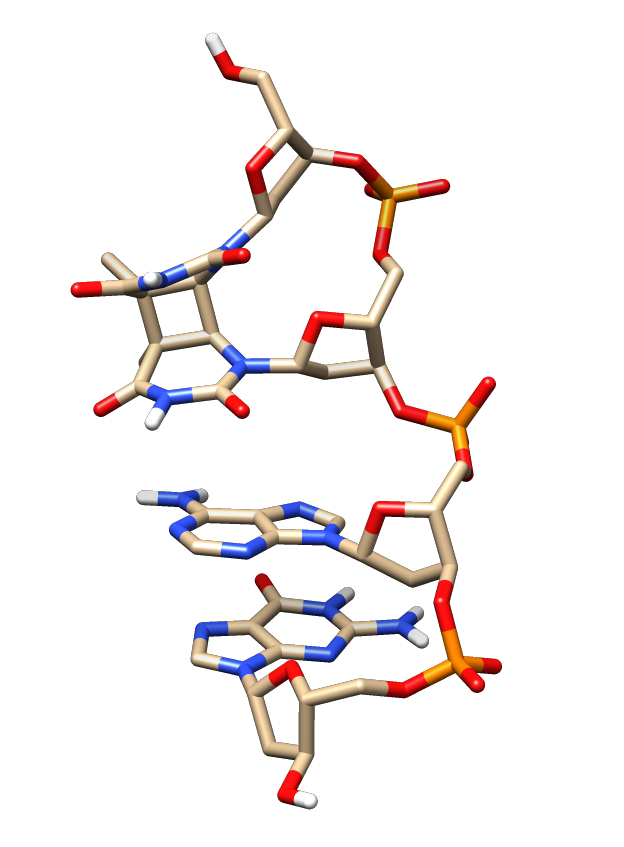

Supplement: SC-015-D3SC04971J-s002 [file SC-015-D3SC04971J-s002.zip › structures/clustering-based-on-MD/ttag_spce/cluster8.png]
